# The highly rearranged mitochondrial genomes of the crabs *Maja crispata* and *Maja squinado* (Majidae) and gene order evolution in Brachyura

**DOI:** 10.1038/s41598-017-04168-9

**Published:** 2017-06-22

**Authors:** Andrea Basso, Massimiliano Babbucci, Marianna Pauletto, Emilio Riginella, Tomaso Patarnello, Enrico Negrisolo

**Affiliations:** 1 University of Padova, Department of Comparative Biomedicine and Food Science (BCA), 35020 Agripolis, Legnaro (PD) Italy; 2 University of Padova, Department of Biology, 35131 Padova, Italy

## Abstract

We sequenced the mitochondrial genomes of the spider crabs *Maja crispata* and *Maja squinado* (Majidae, Brachyura). Both genomes contain the whole set of 37 genes characteristic of Bilaterian genomes, encoded on both α- and β-strands. Both species exhibit the same gene order, which is unique among known animal genomes. In particular, all the genes located on the β-strand form a single block. This gene order was analysed together with the other nine gene orders known for the Brachyura. Our study confirms that the most widespread gene order (BraGO) represents the plesiomorphic condition for Brachyura and was established at the onset of this clade. All other gene orders are the result of transformational pathways originating from BraGO. The different gene orders exhibit variable levels of genes rearrangements, which involve only tRNAs or all types of genes. Local homoplastic arrangements were identified, while complete gene orders remain unique and represent signatures that can have a diagnostic value. Brachyura appear to be a hot-spot of gene order diversity within the phylum Arthropoda. Our analysis, allowed to track, for the first time, the fully evolutionary pathways producing the Brachyuran gene orders. This goal was achieved by coupling sophisticated bioinformatic tools with phylogenetic analysis.

## Introduction

The true crabs belong to Brachyura, the largest clade (an infraorder) of the crustacean Decapoda order (Crustacea, Malacostraca)^1^. Shrimps, prawns, crayfishes and lobsters, some of the most popular crustaceans, are also contained in Decapoda^1^. Currently, more than 7,250 species belong to the Brachyura^2^. Crabs form a big taxonomic group and exhibit a broad array of forms and adaptations, what make them one of the key group to study important biological and evolutionary issues^3^. Several Brachyuran species play an important role as food source for humans and have a relevant commercial value in the fish markets worldwide^4^.

**Figure 1:**
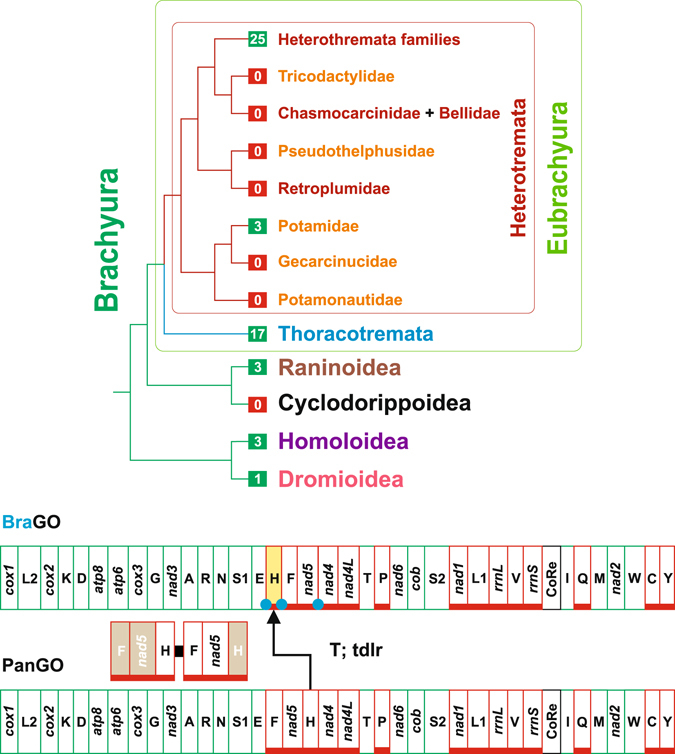
Phylogenetic relationships among major Brachyuran clades and BraGO vs PanGO. The tree depicts the phylogenetic relationships among the major Brachyura lineages cited in the main text. The values in the green/red box refer to the full-length mtDNAs available for that lineage. The taxa names are coloured according to the current placement of taxa themselves within the major Brachyuran groups (*e*.*g*. all families belonging to fresh water crabs are in orange). The genomic transformation from PanGO to BraGO is provided below the tree. PanGO is linearized starting from *cox1*. The genes encoded on the α-strand (orientation from right to left in Fig. 1) are green-boxed, while those encoded on the β-strand (orientation from left to right in Fig. 1) are underlined and red-boxed. Nomenclature: *atp6* and *atp8*: ATP synthase subunits 6 and 8; *cob*: apocytochrome b; *cox1-3*: cytochrome c oxidase subunits 1–3; *nad1-6* and *nad4 L*: NADH dehydrogenase subunits 1–6 and 4 L; *rrnS* and *rrnL*: small and large subunit ribosomal RNA (rRNA) genes; X: transfer RNA (tRNA) genes, where X is the one-letter abbreviation of the corresponding amino acid, in particular L1 (CTN codon family) L2 (TTR codon family), S1 (AGN codon family) S2 (TCN codon family); CoRe: Control Region. T: transposition event. Tdrl: tandem duplication random loss mechanism producing the observed rearrangement. *TrnH*, that changed its position relative to PanGO, through a transposition event, is shown with a yellow background. Conversely, the passively-shifted genes are figured with their original background (see Methods section). The extra copy of every gene that is lost in the genomic rearrangement is figured with a light brown background. A blue circle marks an intergenic spacer present in a position associated to genomic rearrangement.

Currently, the Brachyura are divided in the five major clades Dromiodea, Homoloidea, Cyclodorippoidea, Raninoidea and Eubrachyura (Fig. 1)^2, 5, 6^. The first two taxa form a monophyletic group, as well as Cyclodorippoidea and Raninoidea (Fig. 1). This latter clade is sister taxon of Eubrachyura, the biggest and most differentiated lineage of crabs, encompassing the vast majority of the species. The Eubrachyura are split in two major groups named Heterotremata and Thoracotremata (Fig. 1). Within the Eubrachyura, the phylogenetic position of the primary-freshwater crabs belonging to the families Gecarcinucidae, Potamidae, Potamonautidae, Pseudothelphusidae and Tricodactylidae, is particularly debated^6^. According to the applied phylogenetic method and the type of analysed characters they have been placed within Heterotremata or Thoracotremata^5, 6^. Notably, in the most recent phylogeny available for Brachyura, the freshwater crabs are included within Heterotremata^5, 6^, thus this arrangement is initially followed in the present paper (Fig. 1).

The mitochondrial genome (mtDNA) of Crustacea is usually a double helical and circular molecule spanning 15–18 kb^7^. The most noftable exception is represented by several Isopoda mtDNAs consisting in a combination of a linear molecule approximately 14 kb long, and a circular molecule, made by two linear molecules connected in a head-to-head arrangement^8, 9^.

The Crustacean mtDNA usually contains 37 genes including 13 protein-encoding genes, 22 tRNAs and the small and large ribosomal RNAs (Fig. 1)^7^. Exceptions exist to this largely prevalent scheme and some genes can be absent^10^, or duplicated^11^.

The mitochondrial genes are encoded on both strands of DNA (hereinafter referred to as the α- and β-strands). Genes can overlap, be adjacent or separated by a variable number of nucleotides (*i*.*e*., intergenic spacers). The major intergenic spacer that is always present is the Control Region (CoRe) harbouring the mtDNA origin of replication^12^. The gene order (GO) is not always conserved and Crustacean mtDNAs exhibit different GOs^10^. With respect to a reference GO, genes can be transposed (*i*.*e*., moved to a different placement on the same strand), inverted (*i*.*e*., moved to the opposite strand), or both inverted and transposed (a combination of the first two events). Mitochondrial genes rearrangements are not completely unveiled, although various models have been proposed^13–17^. A transposition can be explained by a tandem duplication and random loss model (tdrl)^13, 14^. A gene inversion is modelled through an intra-mitochondrial recombination^15^, while the inverted transposition can be described through the combination of these two mechanisms. A tandem duplication and random loss (TDRL) event can be applied to analyse the global rearrangement pattern^16, 17^. According to Bernt and Middendorf^17^, TDRL involves a tandem duplication of a continuous segment of genes such that the original segment and its copy are placed consecutively and followed by the loss of one copy of each redundant genes. Multiple genes simultaneously change their position in a TDRL event.

Different GOs have proven to be highly diagnostic in defining animal groups at various taxonomic ranks^18^. In particular, the sister-taxon relationship between Crustacea and Hexapoda (*i*.*e*., the clade Pancrustacea) is strongly supported by their exclusively shared GO (hereafter named PanGO) (Fig. 1)^19^.

The crab mtDNA usually contains the whole set of genes mentioned above. A peculiar condition is found in the potamid crab *Geothelphusa dehani*, which exhibits a case of tRNA remoulding (also named recruitment)^20^. In this case, a point mutation in the anticodon (TAG → TAA) transformed an extra-copy of *trnL1* in a functional *trnL2*, while the true *trnL2* was lost. The remoulding of tRNA is a general process, occurring sparsely in both Eukaryota and Prokaryota, which can involve multiple tRNAs^21–24^. Within Decapoda mtDNAs, the tRNA remoulding has been recorded in species of hermit crabs (Anomura)^22^, as well as in mud shrimps (Gebiidea and Axiidea)^25^. However, in these taxa the point mutation (TAA → TAG) occurred in an extra copy of *trnL2*, which became a functional *trnL1*, while the true *trnL1* is no longer present.

Crabs exhibit different mtDNA GOs, none identical to PanGO^20, 26–33^ (Supplementary Table S1). The most common, the Brachyuran basic GO (hereafter named BraGO)^26, 34^, is depicted in Fig. 1. BraGO differs from PanGO for the transposition of *trnH*, which is located between *trnE* and *trnF*, instead of its placement downstream to *nad5* in PanGO (Fig. 1). Currently, full-length mtDNAs are available for representatives of all major Brachyuran clades^20, 27, 28, 32, 33, 35–41^ (Fig. 1) (see also Supplementary Table S1). However, the taxon coverage is very sparse and the sequencing of new genomes is a high priority. To improve our knowledge on Brachyuran mtDNAs we sequenced the complete genomes of the two spider crabs *Maja crispata* and *Maja squinado* (Majidae). The mtDNAs of both species exhibit the same GO (hereafter MajGO), which is different from any other known animal GO and very re-arranged with respect to PanGO and BraGO. The MajGO is described in details in the Results and Discussion section. After describing the MajGO, we downloaded all complete, or near complete, Brachyuran mtDNAs available in GenBank (release 30.09.2016) and analysed them, in combination with the newly sequenced *Maja* genomes. The goals of present paper were (a) to establish the transformational pathways that led to the diverse GOs observed in Brachyura; (b) to identify the plesiomorphic condition among Brachyuran GOs; (c) to trace the evolutionary steps that produced each unique GO; (d) to test the value of GOs as molecular signatures for the Brachyuran clades.

## Methods

### Ethics statement

No specific permits were required for the work described here. Individuals included in the present study were bought in a fish market or directly collected by one of the authors and they were not subjected to any experimental manipulation. The study was performed in accordance with the EU directive 2010/63/EU and Italian DL 2014/26. The experiments, as well as the euthanasia procedure, were monitored and carried out by authorized staff to minimise animals’ suffering.

### Sampling of Maja crispata and Maja squinado

The specimen of *M*. *crispata* used in the present study was collected by Emilio Riginella in the Venice Lagoon (Italy). The specimen of *M*. *squinado*, caught in the North Adriatic Sea, was acquired in the fish marked of Chioggia (Italy) by Enrico Negrisolo. The samples were preserved in pure ethanol at 4 °C until DNA extraction.

Total DNA was extracted using the ZR Genomic DNA-Tissue Midiprep (Zymo Research corp.) Kit. DNA quality was assessed through electrophoresis. The DNA concentration was determined using the (high sensitivity) Qubit DNA quantification kit (Invitrogen, USA).

### Mitochondrial genome sequencing

The total DNAs, at a concentration of at least 100 ng/µl, were sent to the IGA Technology facility (http://www.igatechnology.com/) (Udine, Italy) to be sequenced using Next-Generation Sequencing (NGS) Illumina HiSeq 2000 and following a 100PE strategy (See the IGA Technology Services for further details on the sequencing strategy). After the sequencing process, 25,946,982 and 32,836,146 paired sequences were obtained for *M*. *crispata* and *M*. *squinado*, respectively.

### Genome assembly and identification of the full length mitochondrial genome

Global assembly of the Illumina reads obtained for *M*. *crispata* and *M*. *squinado* was accomplished with the software CLC Genomics Workbench v8.5 (http://www.clcbio.com). After a BLAST search against the non-redundant database available at the NCBI web site^42^, the sequences that had a high score match with mitochondrial genes (E 10^−20^) were fully annotated using the strategy described in the next section. Afterwards, a single sequence for both *M*. *crispata* and *M*. *squinado* covering at least 95% of the final full length mtDNA (see below), was selected as the template for successive assembly performed using the MITObim program^43^. This second analysis provided a final assembly encompassing the full length mitochondrial genome (mtDNA) for both *M*. *crispata* and *M*. *squinado* Statistics on the final assemblies were calculated with CLC Genomics Workbench v8.5.

The full length sequences of both mtDNAs can be accessed from the EBI/GenBank (*M*. *crispata*, KY650651; *M*. *squinado*, KY650652).

### Mitochondrial genome annotation

The nomenclature of genes and strands are according to Negrisolo *et al*.^44^. The names used to indicate strands are very variable in mtDNA literature^7, 10, 20, 26, 27, 30, 34, 36, 44^. In this paper, the strand encoding the majority of genes is listed as α-strand^20, 34, 44^. First/majority/plus/Heavy (H) -strand are alternative names for the α-strand^7, 10, 26, 27, 30, 36^. Conversely, the strand encoding the minority of genes is listed here as β-strand. Second/minority/minus/Light (L) -strand are alternative names for the β-strand^7, 10, 26, 27, 30, 36^. Initially, the mtDNA sequence was translated into putative proteins using the Transeq program available on the EBI website (https://www.ebi.ac.uk/Tools/st/emboss_transeq/). The identity of these polypeptides was verified using the BLAST program^42^ available at the NCBI website. The boundaries of genes were determined as follows: the 5′ ends of protein-coding genes (PCGs) were defined as the first legitimate in-frame start codon (ATN, GTG, TTG, GTT) in the open reading frame (ORF) that was not located within an upstream gene encoded on the same strand. The only exceptions were *atp6* and *nad4*, which overlap with their upstream genes (*atp8* and *nad4L*, respectively) in many mtDNAs^45^. The PCG terminus was defined as the first in-frame stop codon that was encountered. When the stop codon was located within the sequence of a downstream gene encoded on the same strand, a truncated stop codon (T or TA) adjacent to the beginning of the downstream gene was designated as the termination codon. This codon was thought to be completed by polyadenylation, thereby producing a complete TAA stop codon after transcript processing. Finally, pairwise comparisons with orthologous proteins were performed using ClustalW^46^ to better define the limits of the PCGs.

Regardless of the real initiation codon, a formyl-Met was assumed to be the starting amino acid for all proteins as previously reported in other mitochondrial genomes^47, 48^.

Transfer RNA genes were identified using the tRNAscan-SE program^49^ or recognised manually as sequences having the appropriate anticodon and capable of folding into the typical cloverleaf secondary structure of tRNAs^45^. The validity of these predictions was further enhanced by comparison, based on multiple alignment and structural information, to published orthologous counterparts.

The boundaries of the ribosomal *rrnL* and *rrnS* genes were determined by comparison to the orthologous counterparts present in the mtDNAs of the Brachyura species already sequenced, as well as structural information implied by direct modelling (data not presented here).

### Data set construction

All partial or complete mtDNAs published or publicly available, used in the present paper, were downloaded from GenBank and re-annotated following the approach described above to produce very high-quality annotations. This approach led us to correct the genes boundaries for several taxa. A more drastic change was done with the re-placement of the CoRe in the mtDNA of the potamid crab *Sinopotamon xiushuiense* (KU042041). According to the annotation provided in GenBank (unpublished, see Supplementary Table S1), the CoRe is located between *trnY* and *rrnL* genes. However, in the re-annotation process of *S*. *xiushuiense* mtDNA we discovered, inside of the intergenic spacer (1,221 bp long) located between *rrnS* and *trnI*, the unique signature AACTTATATTACCTA(AT)_27_, which is shared by the CoRe of *G*. *dehani*, the other potamid crab of our data set. Thus, in the present study, this spacer is considered as the true CoRe of *S*. *xiushuiense* mtDNA. Two additional evidences support our choice. First, the CoRe is located between *rrnS* and *trnI* in most of the GOs observed in Brachyura (see below) and more in general in Arthropoda. Secondly, peculiar signatures similar to that presented here are known for other groups of Pancrustacea. For example, the ATAGA(T)_n_ (n > 10) motif characterizes the vast majority of CoRes in the mtDNAs of Lepidoptera^50^.

The availability of new mtDNA sequences in GenBank is a continuous evolving process, occurring at an unpredictable pace. More than fifty partial or complete mtDNAs of species belonging to the major lineages of Brachyura were available in GenBank at the first September 2016. For 50 mtDNAs (partial or complete) it was possible to unambiguously determine their complete GOs, and use them in the full array of analyses presented in this paper. Six outgroups belonging to the infraorders Anomura, Axiidea and Gebiidea (Decapoda, Crustacea) were added to the final set that contains 56 Taxa (T56) (Supplementary Table S1). The mtDNAs of *Huananpotamon lichuanense*, and *Sesarma neglectum* became available too late in GenBank to be fully considered (Supplementary Table S1). Therefore, they were included only in some analyses.

### Multiple alignments of orthologous genes and proteins

Initially each set of the 13 orthologous protein-coding genes, derived from the 56 mtDNAs (Supplementary Table S1), was aligned using the ClustalW program implemented in the MEGA 5.2.2. program^51^. Each alignment was performed with the option “Codons” activated, which ensures that the alignment of DNA sequences is obtained using as backbone the multiple alignment derived from the amino acid counterparts. Following recent findings provided by Tan *et al*.^52^ we did not filter alignments to select blocks of conserved positions, because this process can produce incorrect, statistically supported, trees^52^. Successively, the 13 alignments were concatenated in two data sets (56 T.DNA and its translated counterpart 56 T.PRO) that were used in the phylogenomic analysis. The 56 T.DNA and 56 T.PRO sets spanned respectively 11,208 and 3,736 positions.

### Statistics of DNA/amino acid sequences

The AT-skew = (A − T)/(A + T) and the GC-skew = (G − C)/(G + C) were computed for the α strand of the Brachyuran mtDNAs in order to evaluate the compositional biases^53^. The base compositions were determined with the EditSeq program from the Lasergene software package (DNAStar, Madison, WI).

The total number of codons present in the mitochondrial protein-coding genes was calculated with the MEGA program. Stop codons were excluded from the calculation, because they are not linked to a tRNA family. Analogously, the start codons were omitted, because different codons determine the same formyl-Met as starting amino acid^47, 48^. The abundance of each codon family was expressed as number of codons per thousand codons (CDSpT). The skews computations as well as other statistical calculations were performed with the spreadsheet Microsoft Excel (Microsoft™).

### Mitochondrial phylogenomics of Brachyura

A preliminary analysis on the phylogenetic information present in 56 T.DNA and 56 T.PRO sets was performed according to the likelihood mapping approach^54^ implemented in the IQ-TREE 1.5.2 program^55^. This analysis revealed that the maximum phylogenetic information was present in the 56 T.PRO set (data not shown). Thus, this set was used in the tree searches described below.

Phylogenetic analyses were performed according to the maximum likelihood (ML) method on the 56 T.PRO data set^56^. The ML trees were computed with the program IQ-TREE 1.5.2^55^. In the tree search analysis 100 independent runs were performed in order to avoid/minimize the possibility to be entrapped in sub-optimal trees. The optimal partitioning scheme as well as best fitting evolutionary models were selected with the program IQ-TREE 1.5.2^55, 57^. The best partitioning/evolutionary models were the following: partition 1 (COX1, COX2, COX3, ATP6, CYTB), model mtZOA + I + G4^58^; partition 2 (ATP8, NAD2, NAD3, NAD6), model mtMAM + I + G4^59^; partition 3 (NAD1, NAD4, NAD4L, NAD5), model mtZOA + F + I + G4^58^. In order to minimize the possibility of long-branch attraction phenomena, 56 T.PRO data set was analysed also according to the empirical profile mixture models (C10-C60)^60^ implemented in the IQ-TREE program. The C10-C60 models are the maximum likelihood counterparts of the CAT model developed for Bayesian analysis^61^. The C10-C60 models were applied alone or in combination with the mtZOA^58^ and mtMAM^59^ substitution matrices. All these analyses provided topologies fully congruent with that obtained from the gamma-based models listed above. However, the C10-C60 approaches required much higher computational times than the gamma-based analyses, making unfeasible to use them in the calculation of bootstrap values. Thus, the gamma-based approach was applied to complete the phylogenetic analyses.

The ultrafast bootstrap test (UFBoot) was performed to assess the robustness of ML tree topology (10,000 replicates)^62^. Alternative topologies were evaluated using the Weighted Shimodaira and Hasegawa and the Almost Unbiased tests^55, 63^.

### Gene order analysis of Brachyuran mitochondrial genomes

#### The pairwise approach using the CREx program

Pairwise-comparisons between different GOs were performed with the CREx program^16^. This software analyses genomic rearrangement pathways using common intervals^16, 17, 64^. A common interval is a subset of genes that appear consecutively in two (or more) GOs being investigated^16^.

The CREx program models rearrangements involving transpositions, inversions, inverse transpositions as well as TDRLs^13–17, 64^. CREx produces transformational pathways in which the common intervals, shared by the pairs of GOs, are preserved in all intermediate steps. Once the whole set of common intervals has been determined for a pair of GOs (*e*.*g*., GO1 and GO2) CREx heuristically identifies the most parsimonious transformational pathways that connect GO1 to GO2 and vice versa. For the reader interested on this topic, a detailed description on the functioning of CREx is provided in an open access paper recently authored by our group^18^.

The number of shared common intervals (NSCI) is a measure that can be used to compare the level of similarity of two GOs. Identical GOs share the maximum NSCI value while highly divergent GOs have low NSCIs. Pairwise NSCI-based similarity values were calculated for the Brachyuran GOs. Given the fundamental role played by the control region CR, this latter was considered also in the computation of NSCI values (see results). In the CREx analyses, the software was allowed to compute up to ten alternative scenarios (option max. alternatives = 10) in every search^65^. The output for the different GO reconstructions was always a single transformational pathway. However, the current version of CREx program, which has a heuristic strategy of search, does not explore all possible alternatives, due to an overwhelming computational complexity that would be required for performing this type of analysis. Thus, CREx preferentially provides a single unique transformational scenario, and computes alternative scenarios only in specific cases. Therefore, the transformational pathway reconstructed by CREx is not the only possible and not necessarily the most parsimonious.

Current knowledge on the molecular mechanisms generating the GO rearrangements is very limited and largely insufficient. Thus, it is necessary to rely on mathematical models, implemented in bioinformatic programs, to identify the more probable transformational pathways generating the GOs. Currently, the CREx program is the most flexible and sophisticated software, available to perform this task. The combinatorial mathematics which is used by CREx is rapidly evolving and a natural lag exists between the formulation of new algorithms and their implementation in the software^66^. What is emerging is that, when the reconstructed pathway implies multiple TDRLs, there is not always the certainty that it is the only plausible scenario^66^. The presence of intergenic spacers, located in the genomic positions involved in TDRLs, is regarded as a first, even if weak, independent evidence supporting the most complex pathways^66^. A more conclusive evidence is supposed to be the presence of remnants of the copies of the genes located in these spacers, which were lost in genomic rearrangements, especially TDRLs^66, 67^.

Intergenic spacers, not linked to rearrangements, are common in animal mtDNAs and exhibit a random genomic distribution. A DNA slippage, during the genome replication, is supposed to be the most common mechanism generating these genomic elements^50^. The spacers produced by DNA slippage have usually, but not always, a small size (20 bases ≤ ). The spacers linked to genomic rearrangements are very variable in size, but often they span from some tens to several hundreds of bases (*e*.*g*. Supplementary Fig. S1). Thus in the most favourable situation, it is possible to identify within these spacers the remnants of extra genes copies^67^. Unfortunately, this expectation is often highly diminished by the fact that the size of the spacers, even the largest ones, is much smaller than that of the initial genomic portions involved in the TDRLs. This empirical evidence implies that, once generated, the spacers are subject to a very rapid shrinking. Even worse, local phenomena of slippage and/or a fast substitution rate can further modify these spacers. Additionally, some rearrangements may have occurred very far in the past, leaving small or no spacers at all. Finally, if the reconstructed evolutionary scenario implies multiple TDRLs, the probability to find large size spacers linked to the earlier events should be low. Thus, identify the remnants of lost genes can be a daunting task, impossible to obtain even if very desirable. Conversely, the co-occurrence of multiple intergenic spacers, with genomic positions congruent with the inferred rearrangement pathway, should generate a distributional pattern difficult to explain in terms of pure chance. If this hypothesis holds, the spacers distributional pattern, easily identifiable, becomes a reasonable support (even if not conclusive) to the transformational pathway inferred by CREx.

The occurrence of intergenic spacers associated to rearrangements was checked for every GO to corroborate the obtained evolutionary scenarios identified by CREx. The presence of the remnants of the genes copies located in the intergenic spacers was tested by pair-wise alignments performed with ClustalW^46^.

#### The phylogenetic approach using the TreeREx program

When multiple and highly variable GOs are analysed, it is necessary to apply the phylogenetic approach, implemented in the program TreeREx for inferring the evolutionary pathways leading to the observed diversity of GOs^64^. A fully bifurcating rooted reference tree is necessary. On this tree the pairwise scenarios computed by CREx are mapped along the branches using TreeREx software that can also infer the putative GOs at the internal nodes. Every node is successively labelled, according to a reliability scale implemented in TreeREx, as (a) consistent node, (b) 1-consistent node, and (c) fallback node. In the TreeREx analysis, the consistent nodes are considered to be the most reliable, the 1-consistent nodes exhibit an intermediate level of certainty, and the fallback nodes have the highest level of uncertainty for what concerns the reconstructed GO. More details on the functioning of TreeREx are provided by^18^.

The TreeREx analysis was performed with default settings, as suggested at the website: -s, *i*.*e*. strong consistency method applied; -w, *i*.*e*. weak consistency method applied; -W, *i*.*e*. parsimonious weak consistency method applied; -o, *i*.*e*. get alternative bp scenario for prime nodes; -m = 0, *i*.*e*. maximum number of inversions + TDRL scenarios considered (http://pacosy.informatik.uni-leipzig.de/185-0-TreeREx.html)^64^. The settings above represent a global strategy to search for alternative rearrangements scenarios. In doing so, every node of the reference phylogenetic tree was defined by a GO, regardless of the certainty level for that node.

Both CREx and TreeRex require that analysed GOs include an identical set of genes. Thus if a gene is lacking in a GO it must be removed also from other genomic arrangements. Similarly, if multiple copies of the same gene are present, only one is retained in GO comparisons.

#### Tracking the changes in gene orders

It is well established that genomic rearrangements are modelled through transformational pathways that minimize the number of genes involved in active movements^18^. In describing the above rearrangement leading to BraGO, we attributed it to the transposition of *trnH* between *trnE* and *trnF* (Fig. 1). We did not consider the alternative scenario implying the repositioning of the *trnF* + *nad5* block between *trnH* and *nad4*, because this alternative hypothesis, involving multiple genes, is a less parsimonious explanation for the appearance of BraGO. Of course, the *trnF* + *nad5* block changed its placement downstream (right) to its original position in PanGO, but this movement is viewed as a passive shift, determined by the upstream (left) transposition of *trnH*.

When a transposition occurs between two adjacent genes, the ambiguity on who is the gene moving is resolved by assuming that the repositioning upstream (left) to the original placement is the transposition event, while the shift downstream (to the right) is the passive effect. This is an arbitrary choice because also the alternative scenario is equally parsimonious. The genes, that changed their placement through an obvious transposition event, are figured with a yellow background in this paper whereas the passively-shifted genes are identified with their original background.

However, it is not always possible to determine if the positions exhibited by the genes after a rearrangement are due to active or passive movements. This situation occurs when several genes are involved in complex patterns of movements, which are modelled by single/multiple TDRLs covering large part of the genome. In these cases, the placement of a gene, upstream or downstream to its position in the reference GO, can be the effect of the active re-positioning of the gene itself or a passive-shift due to the movements of the surrounding genes, or a combination of both. Thus, we label with a light-blue background all the genes involved in a repositioning that cannot be identified unambiguously as the result of an active or passive movement. Within this framework, the common intervals, encompassing two or more genes, shared by the re-arranged genomic portion and the reference GOs, are marked with an upper light-blue bar.

## Results and Discussion

### The mtDNAs of *Maja crispata* and *Maja squinado*

**Figure 2:**
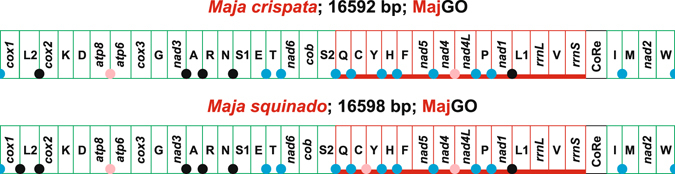
The mitochondrial genomes of *Maja crispata* and *Maja squinado*. The MajGO gene order is depicted and linearized starting from *cox1*. Graphical representation of the mtDNAs and nomenclature of genes as in Fig. 1. A blue circle marks an intergenic spacer assumed to be linked to the genomic rearrangement. A black circle marks an intergenic spacer supposed to be the result of a DNA slippage, during the genome replication. A pink circle marks an overlap between adjacent genes. The α-strand genes are green-boxed, while those of β-strand are underlined and red-boxed.

In this study, the complete mtDNAs of the spider crabs *M*. *crispata* and *M*. *squinado* were sequenced and annotated. The final assembly of *M*. *crispata* was 16,592 bp long and contained 24,734 reads. Additional statistics for the *M*. *crispata* mtDNA were: base coverage = 100%; mismatch = 0%; average coverage depth = 147.25; maximum coverage depth = 409. The final assembly of *M*. *squinado* was 16,598 bp long and contained 20,432 reads. The other statistics for the *M*. *squinado* assembly were: base coverage = 100%; mismatch = 0%; average coverage depth = 121.50; and the maximum coverage depth was 227. The mtDNAs of *M*. *crispata* and *M*. *squinado* contain the full set of 37 genes found in metazoan mtDNAs (Fig. 2). Both mtDNAs present intergenic spacers of variable size (Fig. 2, Supplementary Fig. S1). The newly determined mtDNAs share the same gene order MajGO, which is different from any other animal GO so far sequenced (Fig. 2). In MajGO, all the genes located on the β-strand form a single block, placed between *trnE* and CoRe. For more details on *Maja* mtDNAs, the reader should refer to the Supplementary Figs S1–S6.

### The phylogeny of Brachyura

**Figure 3:**
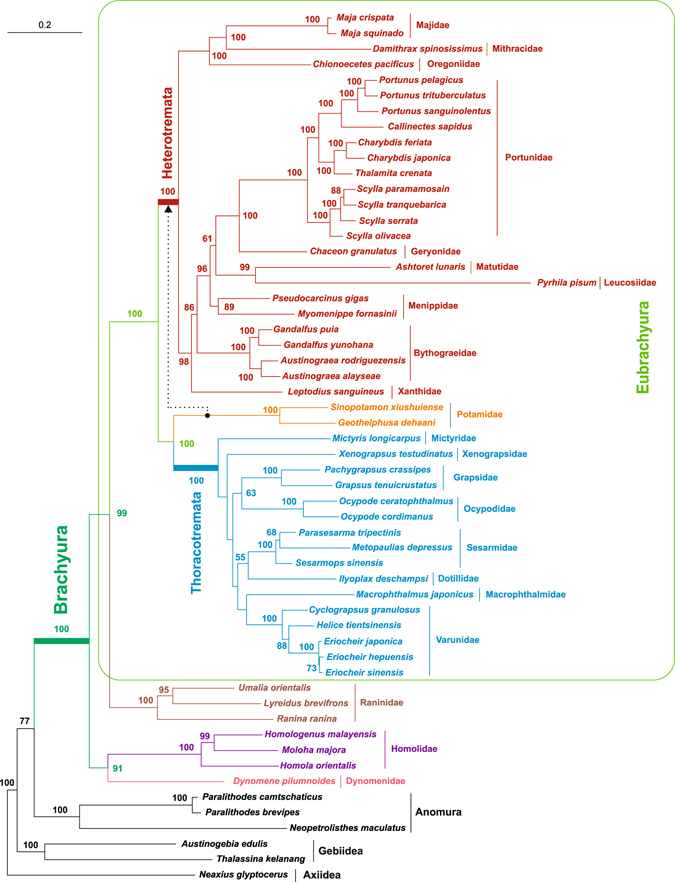
The mitochondrial phylogenomic tree of Brachyura. ML tree (-ln = 112327.8522) obtained from the analysis performed on the 56 T.PRO multiple alignment. Ultrafast bootstrap values ≥ 50% are provided for each node. The alternative phylogenetic placement of Potamidae (see main text) is depicted as a dotted line. The scale bar represents 0.2 substitution/site. Taxa names are coloured as in Fig. 1.

The mitochondrial phylogenomics of Brachyura obtained from 56 T.PRO set is shown in Fig. 3. The majority (39 up to 50) of the nodes of the tree receive good statistical support (UFBoot values > 90%). Only three basal nodes of the Thoracotremata clade does not receive any statistical support (UFBoot values < 50%). The overall phylogenetic outputs are in agreement with those presented in Fig. 1. *Dynomene pilumnoides* (Dromioidea) and the Homolidae species (Homoloidea) cluster together. The Raninidae taxa (Raninoidea) are sister group of Eubrachyura. Within this clade, Heterotremata and Thoracotremata are monophyletic groups receiving very strong statistical support. Taking into account the different taxon sampling, the relationships of the species included within Heterotremata and Thoracotremata are in agreement with the most complete phylogeny of Brachyura, which is based on nuclear genes^5^. Likewise, the superfamilies Ocypodoidea and Grapsoidea do not form monophyletic groups, in perfect agreement with Tsang *et al*.^5^. The only point of strong disagreement is the placement of Potamidae, which is sister group of Thoracotremata in Fig. 3, while is nested within the Heterotremata in the nuclear genes-based phylogeny (Fig. 1). The alternative placement of Potamidae as sister group of Hetereotremata was tested (Fig. 3). However, this relationship is rejected (p-value < 0.01) by both Weighted Shimodaira and Hasegawa and Almost Unbiased tests^63^. The placement of Potamidae shown in Fig. 3 perfectly agrees with the results obtained by other authors working with mitochondrial sequences (DNA and/or proteins)^33, 68^. The placement of primary freshwater crabs, Potamidae and other families, remains a contentious issue^5, 6^. The sparse taxon sampling of mtDNAs does not allow to fully disentangle this problem and a broadening of the taxonomic coverage is a high priority task of future research. A wider taxon and gene sampling will help to ascertain if the discrepancies are due to the nature of the markers, to the different taxon coverage, to the inadequacy of the phylogeny reconstruction algorithms currently available, or a combination of these factors.

The TreeREx program requires a fully resolved tree to reconstruct the evolutionary pathways producing the Brachyuran GO diversity. Initially both the alternative topologies depicted in Fig. 3 were considered. Given that the global evolutionary scenarios do not change for the largest majority of the nodes, the results presented below are referred exclusively to the tree obtained from the analysis of 56 T.PRO set.

### The evolution of mitochondrial gene order in Brachyura

#### The global pattern

**Figure 4:**
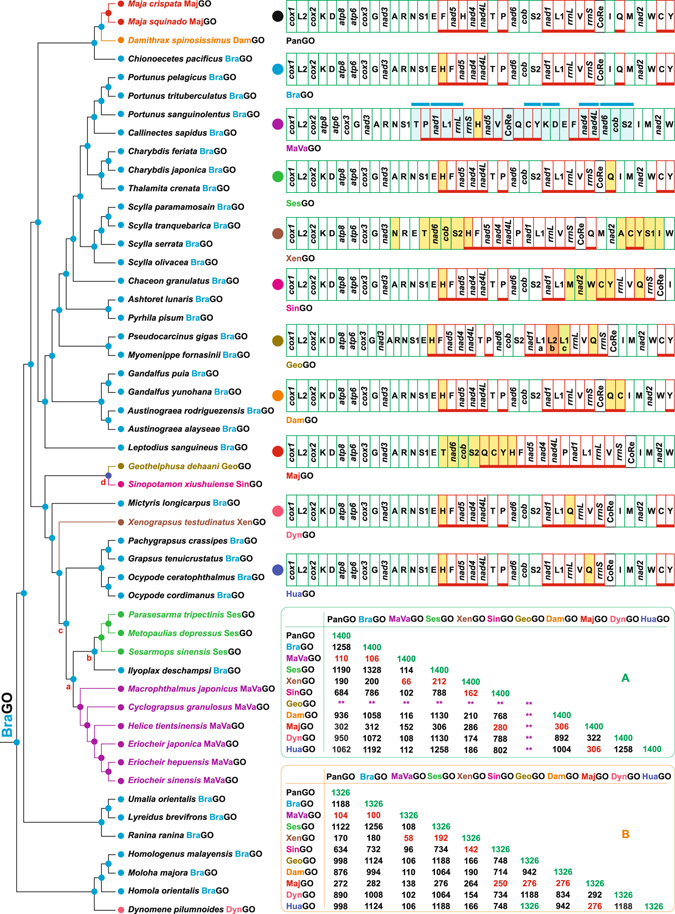
The evolution of mitochondrial gene orders in Brachyura. For most of the nodes the GO was inferred directly with the program TreeREx. For nodes a–d, the GO assignment was performed manually (see main text). L2b, derived from a tRNA remoulding process, not orthologous to true *trnL2s*. L1c, extra copy of *trnL1* not considered in the TreeREx analysis. Table A, NSCI values computed with CREx program through pairwise-comparisons of complete GOs. Table B, NSCI values computed through pairwise-comparisons of GO deprived of L2s. **NSCI values not computed (see main text). The genomic and genetic nomenclature, as well as the colour scheme, are the same as in Fig. 1. The genes that changed their position relative to PanGO, through a transposition event, are shown with a yellow background. The passively-shifted genes are figured with their original background. The genes involved in a repositioning, which cannot be identified unambiguously as the result of a transposition or a passive shift, are figured with a light blue background. In this latter case, the common intervals, encompassing two or more genes, shared by the re-arranged GO with PanGO, are highlighted with a light blue bar.

Currently ten different mtDNA GO are known for Brachyura (Fig. 4, Supplementary Fig. S7). The most widespread is BraGO (Figs 1 and 4). Six of the other GOs are restricted to single species (*i*.*e*., *Dynomene pilumnoides* DynGO; *Damithrax spinosissimus*, DamGO; *Geothelphusa dehaani*, GeoGO; *Huananpotamon lichuanense*, HuaGO; *Sinopotamon xiushuiense* SinGO; *Xenograpsus testudinatus* XenGO). MajGO is shared by both species of *Maja* sequenced within the present study. Finally, SesGO is shared by the crabs belonging to Sesarmidae, while MaVaGO is found in Macrophthalmidae and Varunidae (Fig. 4). The mapping of GOs shows that BraGO occurred at the onset of Brachyura clade. Taking into account that the oldest fossil crabs are known from early Jurassic^69^, BraGO appeared 200 MYA, and since then it has remained unchanged for many Brachyuran taxa (Fig. 4). BraGO shares 1,258 out of 1,400 common intervals with PanGO (Fig. 4). The other Brachyuran GOs, which have evolved from BraGO, can be roughly divided in three groups: (a) very re-arranged GOs (*i*.*e*. MajGO, MaVaGO, and XenGO), which share 312 or less common intervals with BraGO; (b) medium re-arranged GOs (SinGO) (NSCI = 732); (c) low re-arranged GOs (DamGO, DynGO, HuaGO and SesGO), which share 1058 or more common intervals with BraGO.

In the low re-arranged GOs only some tRNAs have changed their placement. Conversely, in both medium and highly re-arranged GOs all types of genes have been involved in the movements. However, none gene was inverted (Fig. 4). The level of rearrangement in various GOs does not appear to be linked, at least in a clearly detectable pattern, to A + T and G + C contents, AT- and GC-skews, as well as codon usage (Supplementary Figs S2–S4). The transformational pathways producing the Brachyuran GOs diversity are described in the following paragraphs.

Initially, the reconstruction of GO evolution was inferred with the TreeREx program (Fig. 4, Supplementary Fig. S7). In particular, TreeREx assigned a GO identical to SesGO to nodes a-c, which exhibited lower consistency values (Supplementary Fig. S7). All species, except *Ilyoplax deschampsi*, deriving from nodes a-b exhibit a CoReQ local arrangement in their mtDNA (Fig. 4), determined by the transposition of *trnQ* immediately downstream to the control region, which is a hotspot of genomic rearrangements^18^. However, the CoReQ arrangement is shared also with DamGO reported in the mtDNA of the unrelated crab *D*. *spinosissimus* (Fig. 4). These findings imply that the transpositions of the mobile *trnQ* generate homoplastic rearrangements in crab GOs. Furthermore, Sesarmidae and Macrophthalmidae + Varunidae, which in Fig. 4 are closely linked, do not result so closely related when a broad taxon sampling exists^5^. Finally, the occurrence of SesGO at nodes a-c implies that two secondary independent reversals to the plesiomorphic condition represented by BraGO occurred in *I*. *deschampsi* mtDNA and in the common ancestor of Grapsidae + Ocypodidae taxa (Figs 3 and 4). Such reversions are extremely improbable events, provided that they happen. Indeed, to our knowledge, they have never been documented in the Bilaterian animals. To complete our reasoning, it must be added here that the accuracy of TreeREx reconstructions is influenced by the coverage of taxon sampling^64^. We regard the output provided by TreeREx program for nodes a-c as an implausible scenario, determined by the sparse taxon sampling coupled with the homoplastic CoReQ arrangement, and influenced by the fact that TreeREx works locally on small subtrees. Thus, in Fig. 4 we manually assign BraGO to nodes a-c. Finally, we manually attributed HuaGO to the node d (Fig. 4). The reasons leading to this assignment are described in details in the paragraph dealing with the evolution of GOs in Potamid crabs.

**Figure 5:**
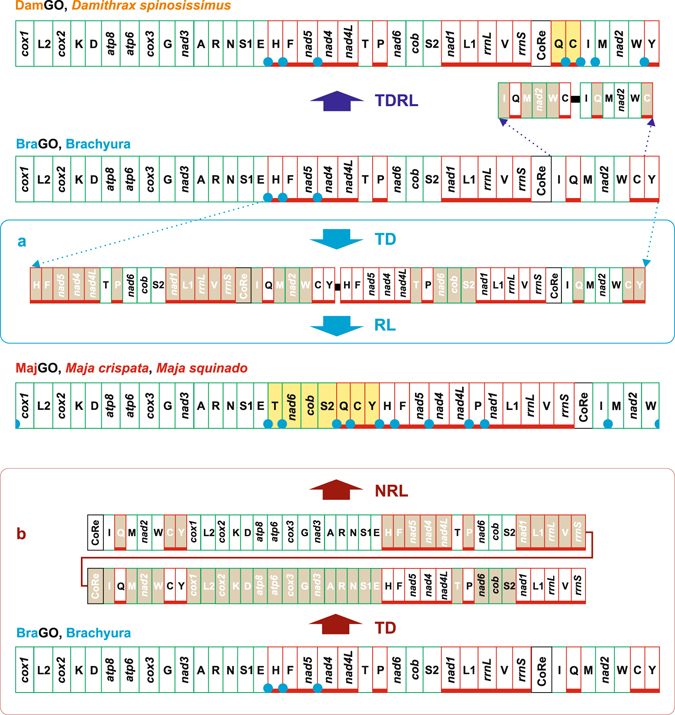
The evolutionary pathways generating DamGO and MajGO arrangements. The rearrangements in the GOs of *D*. *spinosissimus* and *Maja* species are investigated and depicted with respect to BraGO. TDRL, TD/RL, tandem duplication/random loss event. TD/NRL tandem duplication/non random loss event. The genomic and genetic nomenclature, as well as the colour scheme, are the same as in Fig. 1. The genes that changed their position relative to PanGO, through a transposition event, are shown with a yellow background. The passively-shifted genes are figured with their original background. A blue circle marks an intergenic spacer present in a position associated to genomic rearrangement.

**Figure 6:**
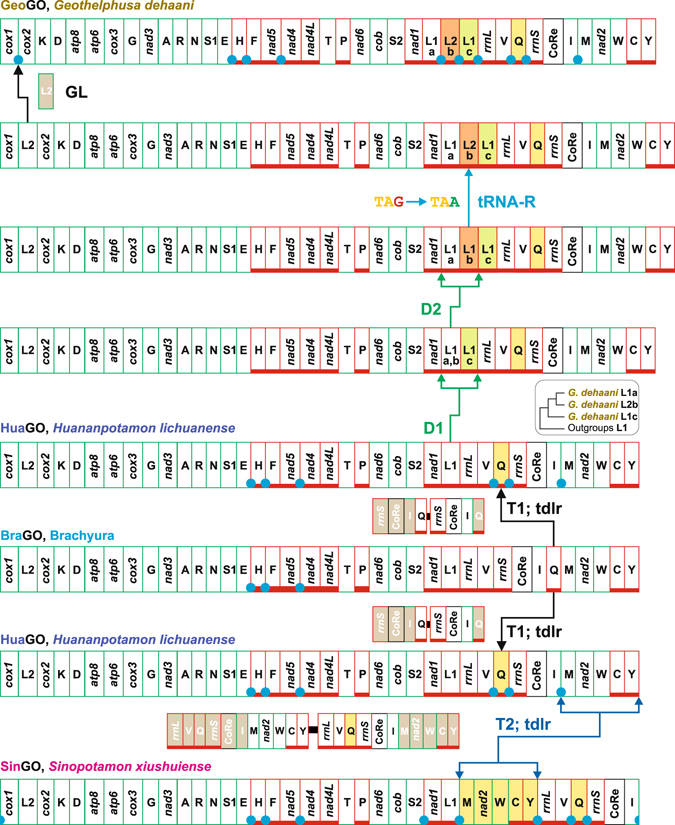
The evolution of GOs in Potamid crabs. Transformational pathways generating GeoGO, HuaGO and SinGO. The rearrangements in the GOs of Potamid species are investigated and depicted with respect to BraGO. T1-T2, transposition events; tdrl, tandem duplication random loss mechanism producing the observed rearrangement; D1-D2, gene duplication events; tRNA-R, tRNA remoulding event; GL, gene loss event. The genomic and genetic nomenclature, as well as the colour scheme, are the same as in Fig. 1. The genes that changed their position relative to PanGO, through a transposition event, are shown with a yellow background. The passively-shifted genes are figured with their original background. A blue circle marks an intergenic spacer present in a position associated to genomic rearrangement.

**Figure 7:**
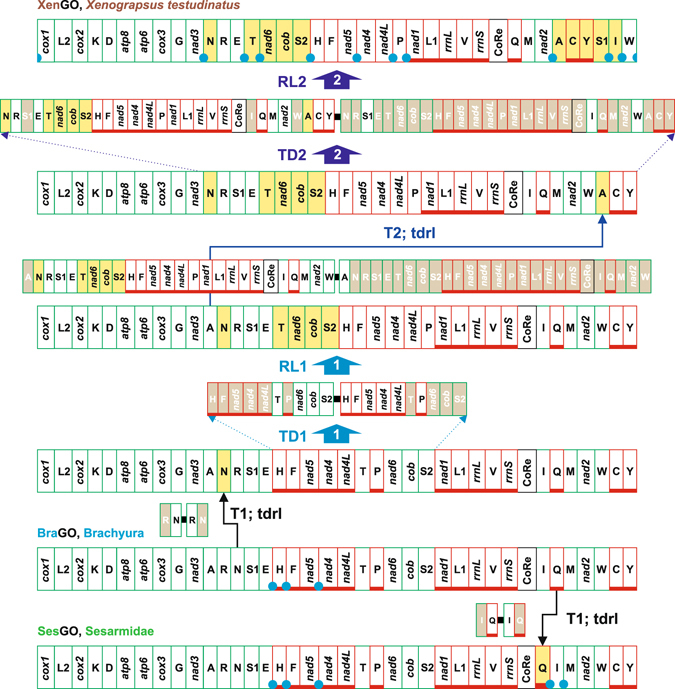
The evolution of GOs in Sesarmidae and *X*. *testudinatus*. The rearrangements in the GOs of Sesarmidae and *X*. *testudinatus* are investigated and depicted with respect to BraGO. T1-T2, transpositions event; tdrl, tandem duplication random loss mechanism producing the observed rearrangement. TD/RL1-2, tandem duplication/random loss events. The genomic and genetic nomenclature, as well as the colour scheme, are the same as in Fig. 1. The genes that changed their position relative to PanGO, through a transposition event, are shown with a yellow background. The passively-shifted genes are figured with their original background. A blue circle marks an intergenic spacer present in a position associated to genomic rearrangement.

**Figure 8:**
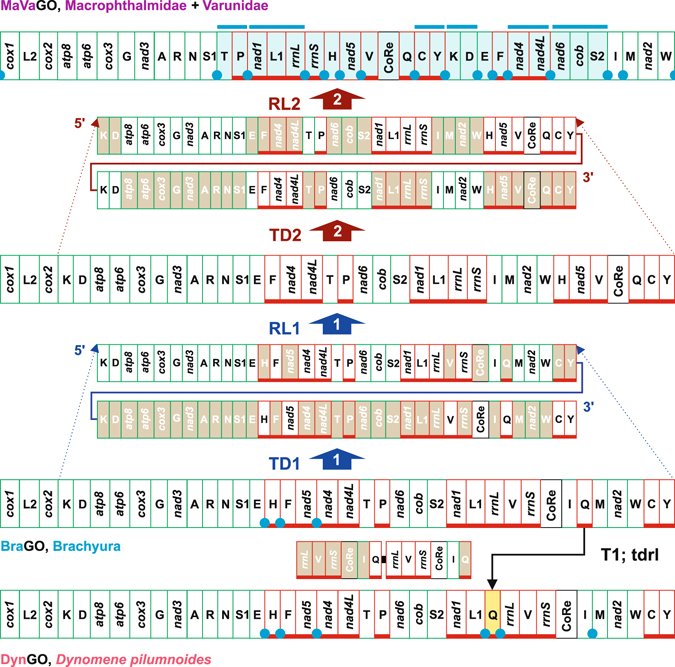
The evolutionary pathways producing the DynGO and MaVaGO arrangements. The rearrangements in the GOs of *D*. *pilumnoides* and Macrophthalmidae + Varunidae are investigated and depicted with respect to BraGO. T1, transposition events; tdrl, tandem duplication random loss mechanism producing the observed rearrangement. TD/RL1-2, tandem duplication/random loss events. The genomic and genetic nomenclature, as well as the colour scheme, are the same as in Fig. 1. The genes that changed their position relative to PanGO, through a transposition event, are shown with a yellow background. The passively-shifted genes are figured with their original background. The genes involved in a repositioning, which cannot be identified unambiguously as the result of a transposition or a passive shift, are figured with a light blue background. In this latter case, the common intervals, encompassing two or more genes, shared by MaVaGO with PanGO, are highlighted with a blue bar. A blue circle marks an intergenic spacer present in a position associated to genomic rearrangement.

All analysed GOs present intergenic spacers located in the genomic positions congruent with the inferred transformational pathways (Figs 5–8)^66^. For most of the GOs, it was possible to identify some possible remnants of genes located within these intergenic spacers (see Methods) (Supplementary Fig. S8). However, in agreement with the expectation expressed in Methods section, the evidence that they are true remnants is often weak. The occurrence of these spacers supports, at minimum, the view that the positions harbouring them played a pivotal role in the transformation pathways, which generated the new GOs. However, provided that alternative transformational routes may involve the same positions and that the current version of CREx reconstructs heuristically preferentially only one scenario, it is not possible to conclude that pathways depicted in Figs 5–8, particularly the most complex ones, represent the only explanation for describing the GO rearrangements. More realistically, each pathway must be regarded as one of the plausible evolutionary scenarios for the studied GO.

#### The evolutionary pathways generating DamGO and MajGO arrangements

The DamGO has been produced through a TDRL event that involved the genomic portion included between *trnI* and *trnC* (Fig. 5). In DamGO, *trnQ* and *trnC* are transposed with respect to BraGO. In the *Maja* species a single TDRL event is necessary to explain the final rearrangement. However, in this latter case the genomic portion involved in the process contains 22 genes plus the CoRe (Fig. 5a). Consequently, the MajGO is the third most re-arranged Brachyuran GO. It shares 312 out of 1,400 common intervals with BraGO (Fig. 4). To our knowledge, MajGO is unique among the animal GOs so far determined, not only for the global placement of the genes, but also for the arrangement in a single unbroken block of the β-strand genes. Alternatively, the MajGO arrangement could be the result of a tandem duplication non-random loss event (TDNRL) (Fig. 5b and Supplementary Fig. S9)^70, 71^. In a TDNRL event, after the duplication of the complete mtDNA, the genes located only on one strand of each duplicated copy are lost. The final GO is strand-biased (further details in Supplementary Fig. S9). Both *Maja* mtDNAs exhibit intergenic spacers (Figs 1 and 5, and Supplementary Fig. S2) at all the positions involved in the TDRL event (Fig. 5a). The remnants of some of genes seem to occur within these spacers (Supplementary Fig. S8), except for *trnE*-*trnT*, which is very short. Both TDRL and TDNRL models implicitly/explicitly allow the presence of intergenic spacers associated to GO rearrangements. Thus, the occurrence of intergenic spacers does not allow to decide what model (TDRL or TDNRL) describes better the evolutionary pathway that generated the *Maja* GO.

#### The evolution of GOs in Potamid crabs

Currently, three full length mtDNAs are available for the Potamid crabs. Their GOs (GeoGO in *G*. *dehaani*; HuaGO in *H*. *lichuanense*; SinGO in *S*. *xiushuiense*; Supplementary Table S1) are distinct and different from BraGO (Figs 4 and 6). GeoGO and SinGO have been included in all our analyses. Due to its late availability, HuaGO has been considered only in the comparisons presented in Figs 4 and 6 and Supplementary Fig. S10. The transformational pathway producing SinGO implies two transpositions (T1-T2) (Fig. 6). T1 generates a GO that is identical to HuaGO of *H*. *lichuanense*. In this case, the CREx exhibits a good predictive capability, because it identified a GO that has a counterpart (HuaGO) in an existing crab. The T2 event produced the transposition of a block of contiguous genes leading to SinGO. BraGO and SinGO shares 786 common intervals (Fig. 4). A partial mtDNA is available in GenBank for the Potamid crab *Sinopotamon yangtsekiense* ^29^, which does not cover some tRNAs, included *trnQ*. In this mtDNA the block *nad2*-*trnY* retains the standard placement observed in BraGO (Fig. 6). However, all genes in the block present multiple frameshift (*nad2*) or mismatches, which severely jeopardize the secondary structure of tRNAs (data not shown). These findings support the hypothesis that this partial mtDNA sequence is not good. Thus new full-length mtDNAs of *S*. *yangtsekiense* and other species of *Sinopotamon* are necessary to study the GO evolution in these Potamid crabs.

A multi-steps strategy was necessary to define the changes leading to GeoGO, due to the peculiar condition exhibited by this genome (Figs 4 and 6, and Supplementary Fig. S10). As mentioned in the introduction, the *G*. *dehaani* mtDNA presents a tRNA remoulding, which involves the *trnL1* and *trnL2*. The true orthologous *trnL2* is lost. Furthermore, two copies of *trnL1* surrounding the functional L2 are found in GeoGO (Figs 4 and 6). The current version of TreeREx program is capable to analyse only GOs that contain an identical set of genes (see Methods). Thus, a first TreeREx search was performed, with a version of GeoGO obtained from the original one by removing *trnL1* (c in Fig. 6). In this analysis, *trnL2* (b) was treated as the true orthologous of the L2 genes present in other GOs (Figs 4 and 6). The TreeREx search allowed to identify the overall scenario presented in Fig. 4, except for nodes a-d. Successively, after removing *trnL2s* (orthologous or functional) from the GOs set, a second analysis was performed with TreeREX (Fig. 4). This reconstruction identified for the node d a GO that is identical to HuaGO deprived of L2 (HuaGO-L2) (Fig. 4). Furthermore, the GeoGO-reduced, a second version of GeoGO deprived of L2 (b) and L1 (c), exhibits an arrangement identical to HuaGO-L2. GeoGO-reduced shares 1,124 out of 1,326 common intervals with BraGO (Fig. 4). As shown above, the first step leading to SinGO was a transposition generating a GO identical to HuaGO (Fig. 6). *G*. *dehaani* and *S*. *xiushuiense* are members of the same phyletic lineage. Given the identical arrangement shared by GeoGO-reduced and HuaGO-L2, and the T1 event observed in the transformational pathway of SinGO, the most parsimonious scenario is to consider the transposition of *trnQ* as the first step shared by the evolutionary changes that produced GeoGO and SinGO (Fig. 6). Thus, combining all the findings presented above, we identified HuaGO as the common first event of the transformational pathways, that generated the GeoGO and SinGO (Fig. 6). HuaGO is considered also the more plausible GO reconstruction for the node d of Fig. 4.

In the evolution of GeoGO, two successive (D1-D2) duplications of *trnL1* followed the transposition T1. These multiplicative steps were the fundament prerequisite for the gene remoulding (GR) process, which generated two functional *trnL2*. The last event was the loss of the true *trnL2*. The deletion of the true *trnL2* could not predate other events. Indeed, *trnL2* is associated to the most numerous codon families not only in Brachyura (see Supplementary Fig. S5) but in animal taxa^50, 67, 72^. It is implausible that the true *trnL2* was lost before the remoulding process occurred.

In an alternative pathway, the D2 duplication could be placed after the remoulding event or after the deletion of the true *trnL2* (Supplementary Fig. S10). In this case a duplication of the tandem genes L1(a)-L2(b) followed by the loss of only the extra copy of L2 (b) is the more plausible scenario. Alternatively, a complicated and improbable event would be necessary, *i*.*e*. the duplication of *trnL2* (b) followed by a back-mutation process leading, through a reverse remoulding, to *trnL1* (c).

The alternative scenario described above, implies that L1 (a) and L1 (c) are sister sequences (Supplementary Fig. S10). However, the phylogenetic analysis^24^ of Brachyuran *trnL1s* reveals that L1 (a) and L1 (c) are not sister sequences (Fig. 6, Supplementary Fig. S10). Conversely, the pathway presented in Fig. 6 is fully consistent with the available data, and represent in our view the most parsimonious and plausible explanation of the GeoGO evolution.

#### The evolution of GOs in Sesarmidae and Xenograpsus testudinatus

SesGO is the least re-arranged among crab GOs as proved by the highest number of shared common intervals (1,328 on 1,400) with BraGO (Fig. 4). The transposition of *trnQ* downstream to CoRe is the molecular signature characterizing all the Sesarmid mtDNAs (Fig. 7). In fact, in addition to the taxa analysed here, SesGO has been described very recently^73^ also for *Sesarma neglectum*, a species whose sequence became available in GenBank too late (2016-10-31) to be analysed here. At opposite, XenGO, is a very modified GO, ranking second among the most re-arranged GOs (Figs 4 and 7). XenGO derived from BraGO through a complex pathway implying two transpositions (*trnN* and *trnA*) and two TDRL events (Fig. 7), computed as the most parsimonious scenario by CREx. These latter involved large blocks of mtDNA in the process of genomic rearrangement (Fig. 7). Intergenic spacers, occurring in several points involved in TDRLs, and possible remnants of genes further favour the XenGO pathway (Fig. 7, Supplementary Fig. S8).

#### The evolutionary pathways producing the DynGO and MaVaGO arrangements

We include in this paragraph the evolutionary reconstructions relative to GOs obtained from distantly related crabs. The choice is dictated simply by practical reasons of presentation of our analysis.

The transformational processes determining the appearance of DynGO are reported in Fig. 8. The transposition of *trnQ* downstream to *trnL1* generated DynGO, which shares 1,008 common intervals with BraGO (Fig. 4). Conversely, MaVaGO is the result of a complex mechanism of rearrangements. Two successive TDRL events involving all 37 genes plus CoRe, with the exclusion of *cox1*, *trnL2* and *cox2*, generated MaVaGO, which characterizes the Macrophthalmidae + Varunidae mtDNAs. The high level of rearrangement is corroborated by the number of common intervals (106) shared by MaVaGO and BraGO. This value is the smallest among the known crab GOs (Fig. 4). Notably, the transformational pathway going from BraGO to MaVaGO, computed as the most parsimonious scenario by CREx, implies only two TDRL events, without an early transposition of *trnQ*, an event hypothesised in the TreeREx reconstruction (see above). The CREx reconstruction further corroborates the manual assignment of BraGO to nodes a-c of Fig. 4. Intergenic spacers, occurring in positions involved in TDLRs, and possible remnants of genes support the MaVaGO scenario (Fig. 8, Supplementary Fig. S8).

### The Brachyuran GOs as molecular signatures

Even restricting the analysis of GOs to the placement of the genes in a linearized genome and ignoring their orientation, the 37 standard animal mitochondrial genes can be arranged in an astonishing number of GOs (*i*.*e*., 37! = 1.367 × 10^43^ or 38! if the CoRe is also included), provided that the movement of every gene is equally probable^74^. However, an always increasing amount of evidence shows that the equally probable movement scenario is totally unrealistic and that some genes are much more mobile than others^18^. In reality, the gene movements in mtDNAs occur preferentially along specific pathways. This characteristic reduces the number of possible arrangements that are likely to be observed, and drastically increases the probability of convergent evolution in GOs. Convergence can be limited to the sharing of local homoplastic rearrangements or involve the full rearrangement of a GO^18^. The tRNAs are the most mobile genes^13, 18, 74^. Furthermore, the genes most prone to homoplastic rearrangements are contiguous in the genome or located around the origin of replication of the mtDNA^18, 75, 76^.

The transposition of *trnH* upstream to *trnF* was the first event of Brachyuran mtDNA. It characterizes still most of the GOs except MaVaGO, where successive rearrangements have disrupted this pairing. *TrnH* is not close to CoRe in PanGO (Figs 1 and 4). Furthermore, the transposition of *trnH* in BraGO, can be described as a long range movement, because it implies the repositioning upstream to the largest coding gene of mtDNA, *i*.*e*. *nad5* (Fig. 1). BraGO is known only for true crabs and our expectation is that this GO represents a strong molecular signature for the Brachyuran clade, in agreement with earlier suggestions^34^.

The analysis of the other Brachyuran GOs reveals that with the exception of the highly re-arranged MajGO, MaVaGO, and XenGO, and the peculiar situation of GeoGO, the remaining GOs have been generated by the transpositions of *trnQ* and *trnC* (Figs 4–8). This hypermobility has generated local homoplastic arrangements. The most widespread is CoReQ, which is shared by DamGO, MaVAGO, SesGO, and XenGO, even if the mechanisms generating this arrangement are different (Figs 5–8). Similarly, a CoReQC homoplastic arrangement occurs in DamGO and MaVaGO (Figs 4–5 and 8).

These findings show that in Brachyura only complete GOs must be considered as molecular signatures, a result mirroring a general behaviour of animal GOs^18^.

MajGO, MaVaGO, and XenGO exhibit high level of rearrangements involving multiple genes (Figs 4–8). The probability of multiple independent appearances of these GOs seems very low. MajGO is shared by *M*. *crispata* and *M*. *squinado*, that are sister species^77^. Thus, our expectation is that MajGO is a synapomorphy characterizing at minimum this subclade of *Maja*, and possibly the whole genus. The range of occurrence of MajGO requires further sequencing efforts. Also, MaVaGO is expected to be a true synapomorphy defining a clade containing all crabs sharing this GO. Finally, XenGO is an apomorphy currently known only for *X*. *testudinatus*.

DynGO, DamGO, HuaGO and SinGO are molecular signatures for taxa possessing them. The low level of rearrangement coupled with the type of genes transposed suggest caution in assigning them the status of mitochondrial genomic apomorphies. The invitation to cautiousness is supported by the increasing evidence that same homoplastic GO can be shared by unrelated animal taxa as recently demonstrated for butterflies, some ants and crickets^18, 78^. Even in these cases the homoplastic GOs represent molecular signatures. However, they cannot be used alone as diagnostic feature exclusively characterizing the taxa possessing them. The homoplastic GOs must be evaluated in a phylogenetic context^18^.

The present study, which is the first to be conducted coupling sophisticated bioinformatic tools with phylogenetic analysis, confirms that the Brachyura are a hot-spot of GOs diversity among Arthropoda. Currently a full length mtDNA is available for less than 1% (52 out of 7250) of the crab species^2^ (Supplementary Table S1). The high number of crab GOs (10) so far determined lead us to suggest that new GOs will be discovered with the increase of the taxon coverage.

## Electronic supplementary material

Basso et al. Supllementary Information
