# Supplementary material for: The highly rearranged mitochondrial genomes of the crabs Maja crispata and Maja squinado (Majidae) and gene order evolution in Brachyura
Source: Sci Rep. 2017 Jun 22;7:4096. doi: 10.1038/s41598-017-04168-9 (PMC5481413; doi:10.1038/s41598-017-04168-9)
Supplement: Supplementary file 1 — Basso et al. Supllementary Information [file 41598_2017_4168_MOESM1_ESM.pdf]

# Supplementary Information

# The highly rearranged mitochondrial genomes of the crabs *Maja crispata* and *Maja squinado* (Majidae) and gene order evolution in Brachyura

**Andrea Basso[1*], Massimiliano Babbucci[1*], Marianna Pauletto[1], Emilio Riginella[2], Tomaso Patarnello[1] & Enrico Negrisolo[1]**

**[1] University of Padova, Department of Comparative Biomedicine and Food Science (BCA), 35020, Agripolis - Legnaro (PD), Italy.**

**[2] University of Padova, Department of Biology, 35131, Padova, Italy.**

**[*] These authors contributed equally to this work.**

**Correspondence and requests for materials should be addressed to E.N.**

**enrico.negrisolo@unipd.it.**

Table S1. List of taxa, taxonomy, accession numbers, references and crab gene orders

| | Infraorder | | | Superfamily | Family | Species | Accession number | GenBank availability | Reference | crab GOs |
|---|---|---|---|---|---|---|---|---|---|---|
| outgroups | **Axiidea** | | | | Strahlaxiidae | *Neaxius glyptocercus* (von Martens, 1868) | JN897379 | 03.12.2012 | P;09 | *** |
| | **Gebiidea** | | | | Upogebiidae | *Austinogebia edulis* (Ngoc-Ho & Chan, 1992) | JN897376 | 03.12.2012 | P;09 | *** |
| | | | | | Thalassinidae | *Thalassina kelanang* Moh & Chong, 2009 | JN897378 | 03.12.2012 | P;09 | *** |
| | **Anomura** | | | | Lithodidae | *Paralithodes brevipes* (H. Milne Edwards & Lucas, 1841) | AB735677 | 28.05.2013 | U;38 | *** |
| | | | | | Lithodidae | *Paralithodes camtschaticus* (Tilesius, 1815) | JX944381 | 22.07.2013 | P;06 | *** |
| | | | | | Porcellanidae | *Neopetrolisthes maculatus* (H. Milne Edwards, 1837) | KC107816 | 20.03.2013 | P;22 | *** |
| 1 | **Brachyura** | | | **Dromioidea** | Dynomenidae | *Dynomene pilumnoides* Alcock, 1900 | KT182070 | 20.08.2016 | P;24 | DynGO |
| 2 | | | | **Homoloidea** | Homolidae | *Homola orientalis* Henderson, 1888 | KT182071 | 30.06.2016 | P;24 | BraGO |
| 3 | | | | | Homolidae | *Homologenus malayensis* Ihle, 1912 | KJ612407 | 31.12.2014 | P;03 | BraGO |
| 4 | | | | | Homolidae | *Moloha majora* (Kubo, 1936) | KT182069 | 25.01.2016 | P;24 | BraGO |
| 5 | | | | **Raninoidea** | Raninidae | *Lyreidus brevifrons* Sakai, 1937 | KM983394 | 15.03.2015 | P;23 | BraGO |
| 6 | | | | | Raninidae | *Ranina ranina* (Linnaeus, 1758) | KM189817 | 26.08.2014 | P;02 | BraGO |
| 7 | | | | | Raninidae | *Umalia orientalis* (Sakai, 1963) | KM365084 | 15.03.2015 | P;23 | BraGO |
| 8 | | **Eubrachyura** | | **Potamoidea** | Potamidae | *Geothelphusa dehaani* (White, 1847) | AB187570 | 08.08.2012 | P;21 | GeoGO |
| 9 | | | | **Potamoidea** | Potamidae | *Sinopotamon xiushuiense* Dai, Zhou & Peng, 1995 | KU042041 | 16.12.2015 | U;35 | SinGO |
| 10 | | | **Thoracothremata** | **Grapsoidea** | Grapsidae | *Grapsus tenuicrustatus* (Herbst, 1783) | KT878721 | 08.03.2016 | P;27 | BraGO |
| 11 | | | | **Grapsoidea** | Grapsidae | *Pachygrapsus crassipes* Randall, 1840 | KC878511 | 11.09.2014 | P;43 | BraGO |
| 12 | | | | **Grapsoidea** | Sesarmidae | *Metopaulias depressus* Rathbun, 1896 | KX118277 | 11.06.2016 | U;08 | SesGO |
| 13 | | | | **Grapsoidea** | Sesarmidae | *Parasesarma tripectinis* (Shen, 1940) | KU343209 | 23.04.2016 | U;18 | SesGO |
| 14 | | | | **Grapsoidea** | Sesarmidae | *Sesarmops sinensis* (H. Milne Edwards, 1834) | KR336554 | 01.05.2016 | U;11 | SesGO |
| 15 | | | | **Grapsoidea** | Varunidae | *Cyclograpsus granulosus* H. Milne Edwards, 1853 | LN624373 | 26.06.2015 | P;29 | MaVaGO |
| 16 | | | | **Grapsoidea** | Varunidae | *Eriocheir hepuensis* Dai, 1991 | FJ455506 | 22.11.2008 | P;36 | MaVaGO |
| 17 | | | | **Grapsoidea** | Varunidae | *Eriocheir japonica* (De Haan, 1835) | FJ455505 | 22.11.2008 | P;36 | MaVaGO |
| 18 | | | | **Grapsoidea** | Varunidae | *Eriocheir sinensis* H. Milne Edwards, 1853 | AY274302 | 18.04.2005 | P;25 | MaVaGO |
| 19 | | | | **Grapsoidea** | Varunidae | *Helice tientsinensis* Rathbun, 1931 | KR336555 | 01.05.2016 | U;11 | MaVaGO |
| 20 | | | | **Grapsoidea** | Xenograpsidae | *Xenograpsus testudinatus* N. K. Ng, Huang & Ho, 2000 | EU727203 | 03.11.2009 | P;05 | XenGO |
| 21 | | | | **Ocypodoidea** | Dotillidae | *Ilyoplax deschampsi* (Rathbun, 1913) | JF909979 | 22.07.2014 | P;04 | BraGO |
| 22 | | | | **Ocypodoidea** | Macrophthalmidae | *Macrophthalmus japonicus* (De Haan, 1835) | KU343211 | 23.04.2016 | U;18 | MaVaGO |
| 23 | | | | **Ocypodoidea** | Mictyridae | *Mictyris longicarpus* Latreille, 1806 | LN611670 | 02.10.2014 | P;31 | BraGO |
| 24 | | | | **Ocypodoidea** | Ocypodidae | *Ocypode ceratophthalmus* (Pallas, 1772) | LN611669 | 02.10.2014 | P;32 | BraGO |
| 25 | | | | **Ocypodoidea** | Ocypodidae | *Ocypode cordimanus* Latreille, 1818 | KT896743 | 08.03.2016 | P;26 | BraGO |
| 26 | | | **Heterotremata** | **Majoidea** | Majidae | *Maja crispata* Risso, 1827 | **KY650651** | **NEW** | P;01 | MajGO |
| 27 | | | | **Majoidea** | Majidae | *Maja squinado* (Herbst, 1788) | **KY650652** | **NEW** | P;01 | MajGO |
| 28 | | | | **Majoidea** | Mithracidae | *Damithrax spinosissimus* (Lamarck, 1818) | KM405516 | 25.10.2014 | P;14 | DamGO |
| 29 | | | | **Majoidea** | Oregoniidae | *Chionoecetes pacificus* Sakai, 1978 | AB735678 | 04.07.2013 | U;38 | BraGO |
| 30 | | | | **Bythograeoidea** | Bythograeidae | *Austinograea alayseae* Hessler & Martin, 1989 | JQ035660 | 11.02.2013 | P;40 | BraGO |
| 31 | | | | **Bythograeoidea** | Bythograeidae | *Austinograea rodriguezensis* Tsuchida & Hashimoto, 2002 | JQ035658 | 11.02.2013 | P;40 | BraGO |
| 32 | | | | **Bythograeoidea** | Bythograeidae | *Gandalfus puia* McLay, 2007 | KR002727 | 21.06.2015 | P;07 | BraGO |
| 33 | | | | **Bythograeoidea** | Bythograeidae | *Gandalfus yunohana* (Takeda, Hashimoto & Ohta, 2000) | EU647222 | 27.10.2010 | P;41 | BraGO |
| 34 | | | | **Calappoidea** | Matutidae | *Ashtoret lunaris* (Forskål, 1775) | LK391941 | 20.06.2014 | P;33 | BraGO |
| 35 | | | | **Eriphioidea** | Menippidae | *Myomenippe fornasinii* (Bianconi, 1851) | LK391943 | 20.06.2014 | P;34 | BraGO |
| 36 | | | | **Eriphioidea** | Menippidae | *Pseudocarcinus gigas* (Lamarck, 1818) | AY562127 | 15.02.2006 | P;17 | BraGO |
| 37 | | | | **Portunoidea** | Geryonidae | *Chaceon granulatus* (Sakai, 1978) | AB769383 | 31.01.2014 | U;39 | BraGO |
| 38 | | | | **Portunoidea** | Portunidae | *Callinectes sapidus* Rathbun, 1896 | AY363392 | 19.05.2005 | P;19 | BraGO |
| 39 | | | | **Portunoidea** | Portunidae | *Charybdis feriata* (Linnaeus, 1758) | KF386147 | 13.08.2015 | P;12 | BraGO |
| 40 | | | | **Portunoidea** | Portunidae | *Charybdis japonica* (A. Milne-Edwards, 1861) | FJ460517 | 01.07.2010 | P;10 | BraGO |
| 41 | | | | **Portunoidea** | Portunidae | *Portunus pelagicus* (Linnaeus, 1758) | KM977882 | 14.01.2015 | P;15 | BraGO |
| 42 | | | | **Portunoidea** | Portunidae | *Portunus sanguinolentus* (Herbst, 1783) | KT438509 | 19.10.2015 | P;16 | BraGO |
| 43 | | | | **Portunoidea** | Portunidae | *Portunus trituberculatus* (Miers, 1876) | AB093006 | 12.07.2003 | P;37 | BraGO |
| 44 | | | | **Portunoidea** | Portunidae | *Scylla olivacea* (Herbst, 1796) | FJ827760 | 31.03.2009 | U;20 | BraGO |
| 45 | | | | **Portunoidea** | Portunidae | *Scylla paramamosain* Estampador, 1949 | FJ827761 | 31.03.2009 | P;13 | BraGO |
| 46 | | | | **Portunoidea** | Portunidae | *Scylla serrata* (Forskål, 1775) | FJ827758 | 31.03.2009 | U;20 | BraGO |
| 47 | | | | **Portunoidea** | Portunidae | *Scylla tranquebarica* (Fabricius, 1798) | FJ827759 | 31.03.2009 | U;20 | BraGO |
| 48 | | | | **Portunoidea** | Portunidae | *Thalamita crenata* Rüppell, 1830 | LK391945 | 20.07.2014 | P;30 | BraGO |
| 49 | | | | **Xanthoidea** | Xanthidae | *Leptodius sanguineus* (H. Milne Edwards, 1834) | KT896744 | 07.04.2016 | P;28 | BraGO |
| 50 | | | | **Leucosioidea** | Leucosiidae | *Pyrhila pisum* (De Haan, 1841) | KU343210 | 23.04.2016 | U;18 | BraGO |
| ! | | | **Thor** | **Grapsoidea** | Sesarmidae | *Sesarma neglectum* de Man, 1887 | KX156954 | **31.10.2016** | P; 43 | SesGO |
| ! | | | | **Potamoidea** | Potamidae | *Huananpotamon lichuanense* Dai, Zhou & Peng, 1995 | KX639824 | **02.10.2016** | U; 44 | HuaGO |

P, published reference; U, Unpublished; GO, Gene order
Species taxonomy based on various sources. In particular, for marine taxa the nomenclature is that found in the WoRMS data base (World Register of Marine Species)
WoRMS Editorial Board (2016). World Register of Marine Species. Available from **http://www.marinespecies.org** at VLIZ. Accessed 2016-09-23. doi:10.14284/170
***, GO not presented here; **BraGO,** Brachyuran basic GO**;** **DamGO**, *Damithrax spinosissimus* GO*;* **DynGO**, *Dynomene pilumnoides* GO*;* **GeoGO**, *Geothelphusa dehaani* GO; **HuaGO,** *Huananpotamon lichuanense* GO, **MajGO**, *Maja* genus GO; **MaVaGO**, Macrophthalmidae + Varunidae GO; **SesGO**, Sesarmidae GO; **SinGO**, *Sinopotamon xiushuiense* GO; **XenGO**, *Xenograpsus testudinatus* GO.
!, this mtDNA became available too late to be incorporated in all the analyses. It was considered only in some analyses (see main text). Thor, Thoracothremata.

## References


1. Basso et al. **this paper**
2. Cheng, J., Jiang W., Shi, H., Sha, Z. The complete mitochondrial genome of red frog crab *Ranina ranina* (Crustacea: Decapoda: Brachyura: Raninidae). *Mitochondrial DNA*. **27**, 1368–1369, doi: 10.3109/19401736.2014.947584 (2016).
3. Hui, M, Liu, Y, Cui, Z. First complete mitochondrial genome of primitive crab *Homologenus malayensis* (Decapoda: Brachyura: Podotremata: Homolidae). *Mitochondrial DNA* **27**, 859–860, doi: 10.3109/19401736.2014.919476 (2016).
4. Ji, Y.K., Wang, A., Lu, X.L., Song, D.H., Jin, Y.H., Lu, J.J., Sun, HY. Mitochondrial genomes of two brachyuran crabs (Crustacea: Decapoda) and phylogenetic analysis. *Journal of Crustacean Biology*. **34**, 494–503, doi: 10.1163/1937240X-00002252 (2014).
5. Ki, J.S., Dahms, H.U., Hwang, J.S., Lee, J.S. The complete mitogenome of the hydrothermal vent crab *Xenograpsus testudinatus* (Decapoda, Brachyura) and comparison with brachyuran crabs. *Comparative Biochemistry and Physiology, Part D*. **4**, 290–299, doi: 10.1016/j.cbd.2009.07.002 (2009).
6. Kim, S., Choi, H.G., Park, J.K., Min, G.S. The complete mitochondrial genome of the subarctic red king crab, *Paralithodes camtschaticus* (Decapoda, Anomura). *Mitochondrial DNA* **24**, 350–352, doi: 10.3109/19401736.2012.760555 (2013).
7. Kim, S.J., Moon, J.W., Ju, SJ. Complete mitochondrial genome of the blind vent crab *Gandalfus puia* (Crustacea: Bythograeidae) from the Tonga Arc. *Mitochondrial DNA Part A* **27**, 2719–2720, doi: 10.3109/19401736.2015.1046162 (2016).
8. Lesny, P., Schubart, C.D., Podsiadlowski, L. **Unpublished**. *Metopaulias depressus*
9. Lin, F.J., Liu, Y., Sha, Z., Tsang, L.M., Chu, K.H., Chan, T.Y., Liu, R., Cui, Z. Evolution and phylogeny of the mud shrimps (Crustacea: Decapoda) revealed from complete mitochondrial genomes. *BMC Genomics* **13**, 631, doi: 10.1186/1471-2164-13-631 (2012).
10. Liu, Y., Cui, Z. Complete mitochondrial genome of the Asian paddle crab *Charybdis japonica* (Crustacea: Decapoda: Portunidae): gene rearrangement of the marine brachyurans and phylogenetic considerations of the decapods. *Mol. Biol. Rep*. **37**, 2559–2569, doi: 10.1007/s11033-009-9773-2 (2010).
11. Liu, Q.N., Tang, B.P. **Unpublished**. *Sesarmops sinensis, Helice tientsinensis*
12. Ma, H., Ma, C., Li, C., Lu, J., Zou, X., Gong, Y., Wang, W., Chen, W., Ma, L., Xia L. First mitochondrial genome for the red crab (*Charybdis feriata*) with implication of phylogenomics and population genetics. *Scientific Reports* **5**, 11524, doi: 10.1038/srep11524 (2015).
13. Ma, H., Ma, C., Li, X., Xu, X., Feng, N., Ma, L. The complete mitochondrial genome sequence and gene organization of the mud crab (*Scylla paramamosain*) with phylogenetic consideration. *Gene* **519**, 120–127, doi: 10.1016/j.gene.2013.01.028 (2013).
14. Márquez, E.J., Hurtado-Alarcón, J.C., Isaza, J.P., Alzate, J.F., Campos, N.H. Mitochondrial genome of the Caribbean king crab *Damithrax spinosissimus* (Lamarck, 1818) (Decapoda: Majidae). *Mitochondrial DNA* (in press); doi:10.3109/19401736.2014.961140 (2016).
15. Meng, X., Jia, F., Liu, P., Li, J. The complete mitogenome of blue swimming crab *Portunus pelagicus* Linnaeus, 1766 (Crustacea: Decapoda: Portunidae), *Mitochondrial DNA Part A* **27**, 2789–2790, doi: 10.3109/19401736.2015.1053068, (2016).
16. Meng, X., Jia, F., Zhang, X., Liu, P., Li, J. Complete sequence and characterization of mitochondrial genome in the swimming crab *Portunus sanguinolentus* (Herbst, 1783) (Decapoda, Brachyura, Portunidae). *Mitochondrial DNA Part A* **27**, 3052–3053, doi: 10.3109/19401736.2015.1063130 (2016).
17. Miller, A.D., Murphy, N.P., Burridge, C.P., Austin, C.M. Complete mitochondrial DNA sequences of the decapod crustaceans *Pseudocarcinus gigas* (Menippidae) and *Macrobrachium rosenbergii* (Palaemonidae). *Marine Biotechnology*. **7**, 339–349, doi: 10.1007/s10126-004-4077-8 (2005).
18. Park,Y.J., Park, C.E., Lee, S.H., Ko, H.S., Shin, J.H. **Unpublished**. *Parasesarma tripectinis, Macrophthalmus japonicus, Pyrhila pisum*
19. Place, A.R., Feng, X., Steven, C.R., Fourcade, H.M., Boore, J.L. Genetic markers in blue crabs (*Callinectes sapidus*) II: complete mitochondrial genome sequence and characterization of genetic variation. *Journal of Experimental Marine Biology and Ecology*. **319**, 15–27, doi:10.1016/j.jembe.2004.03.024 (2005).
20. Sangthong, P. **Unpublished**, *Scylla olivacea, Scylla serrata, Scylla tranquebarica*
21. Segawa, R.D., Aotsuka, T. The mitochondrial genome of the Japanese freshwater crab, *Geothelphusa dehaani* (Crustacea: Brachyura): evidence for its evolution via gene duplication. *Gene* **355**, 28–39, doi: 10.1016/j.gene.2005.05.020 (2005).
22. Shen, H., Braband, A., Scholtz, G. Mitogenomic analysis of decapod crustacean phylogeny corroborates traditional views on their relationships. *Molecular Phylogenetics and Evolution*. **66**, 776–789, doi: 10.1016/j.ympev.2012.11.002 (2013).
23. Shi, G., Cui, Z., Hui, M., Liu, Y., Chan, T.Y., Song, C. The complete mitochondrial genomes of *Umalia orientalis* and *Lyreidus brevifrons*: The phylogenetic position of the family Raninidae within Brachyuran crabs. *Marine Genomics*. **21**, 53–61, doi: 10.1016/j.margen.2015.02.002 (2015).
24. Shi, G., Cui, Z., Hui, M., Liu, Y., Chan, T.Y., Song, C. Unusual sequence features and gene rearrangements of primitive crabs revealed by three complete mitochondrial genomes of Dromiacea. *Comparative Biochemistry and Physiology - Part D: Genomics and Proteomics* **20**, 65–73, doi: 10.1016/j.cbd.2016.07.004 (2016).
25. Sun, H., Zhou, K., Song, D. Mitochondrial genome of the Chinese mitten crab *Eriocheir japonica sinenesis (*Brachyura: Thoracotremata: Grapsoidea) reveals a novel gene order and two target regions of gene rearrangements. *Gene* **349**, 207–217, doi: 10.1016/j.gene.2004.12.036 (2005).
26. Sung, J.M., Lee, J.H., Kim, S.G., Zafer Karagozlu, M., Kim, C.B. Analysis of complete mitochondrial genome of *Ocypode cordimanus* (Latreille, 1818) (Decapoda, Ocypodidae), *Mitochondrial DNA Part B* **1**, 363–364, doi: 10.1080/23802359.2016.1168718 (2016).
27. Sung, J.M., Lee, J.H., Kim, S.K., Zafer Karagozlu, M., Kim, C.B. The complete mitochondrial genome of *Grapsus tenuicrustatus* (Herbst, 1783) (Decapoda, Grapsidae). *Mitochondrial DNA Part B* **1**, 441–442, doi: 10.1080/23802359.2016.1180559 (2016).
28. Sung, J.M., Lee, J.H., Kim, S.G., Zafer Karagozlu, M., Kim, C.B. Complete mitochondrial genome of *Leptodius sanguineus* (Decapoda, Xanthidae). *Mitochondrial DNA Part B* **1**, 500–501, doi: 10.1080/23802359.2016.1192505 (2016).
29. Tan, M.H., Gan, H.M., Lee, Y.P., Austin, C.M. The complete mitogenome of purple mottled shore crab *Cyclograpsus granulosus* H. Milne-Edwards, 1853 (Crustacea: Decapoda: Grapsoidea). *Mitochondrial DNA* (in press), doi:10.3109/19401736.2014.989514 (2016).
30. Tan, M.H, Gan, H.M., Lee, Y.P., Austin, C.M. The complete mitogenome of the swimming crab *Thalamita crenata* (Rüppell, 1830) (Crustacea; Decapoda; Portunidae). *Mitochondrial DNA Part A* **27**, 1275–1276, doi: 10.3109/19401736.2014.945553 (2016).
31. Tan, M.H., Gan, H.M., Lee, Y.P., Austin, C.M. The complete mitogenome of the soldier crab *Mictyris longicarpus* (Latreille, 1806) (Crustacea: Decapoda: Mictyridae). *Mitochondrial DNA Part A* **27**, 2121–2122, doi:10.3109/19401736.2014.982585 (2016).
32. Tan, M.H., Gan, H.M., Lee, Y.P., Austin, C.M. The complete mitogenome of the ghost crab *Ocypode ceratophthalmus* (Pallas, 1772) (Crustacea: Decapoda: Ocypodidae). *Mitochondrial DNA Part A* **27**, 2123–2124, doi:10.3109/19401736.2014.982587 (2016).
33. Tan, M.H., Gan, H.M., Lee, Y.P., Austin, C.M. The complete mitogenome of the moon crab *Ashtoret lunaris* (Forskal, 1775), (Crustacea; Decapoda; Matutidae). *Mitochondrial DNA Part A* **27**, 1313–1314, doi: 10.3109/19401736.2014.945572 (2016).
34. Tan, M.H., Gan, H.M., Lee, Y.P., Austin, C.M. The complete mitogenome of the stone crab *Myomenippe fornasinii* (Bianconi, 1851) (Crustacea: Decapoda: Menippidae). *Mitochondrial DNA Part A* **27**, 1374–1375, doi: 10.3109/19401736.2014.947587 (2016).
35. Wang, Y., Zhou, X., Xu, W., Bai, J., Zeng, Q. **Unpublished**. *Sinopotamon xiushuiense*
36. Wang, J., Huang, L., Cheng, Q., Lu, G., Wang. C. Complete mitochondrial genomes of three mitten crabs, *Eriocheir sinensis*, *E. hepuensis*, and *E. japonica*. *Mitochondrial DNA Part A* **27**, 1175–1176, doi: 10.3109/19401736.2014.936425 (2016).
37. Yamauchi, M.M., Miya, M.U., Nishida, M. Complete mitochondrial DNA sequence of the swimming crab, *Portunus trituberculatus* (Crustacea: Decapoda: Brachyura). *Gene* **311**, 129–135, doi: 10.1016/S0378-1119(03)00582-1 (2003).
38. Yanagimoto,T., Kobayashi, T. **Unpublished**. *Paralithodes brevipes, Chionoecetes pacificus*
39. Yanagimoto,T. **Unpublished**. *Chaceon granulatus*
40. Yang, J.S., Lu, B., Chen, D.F., Yu, Y.Q., Yang, F., Nagasawa, H., Tsuchida, S., Fujiwara, Y., Yang, W.J. When did decapods invade hydrothermal vents? Clues from the Western pacific and Indian oceans. *Molecular Biology and Evolution*. **30**, 305–309, doi: 10.1093/molbev/mss224 (2013).
41. Yang, J.S., Nagasawa, H., Fujiwara, Y., Tsuchida, S., Yang, W.J. The complete mitogenome of the hydrothermal vent crab *Gandalfus yunohana* (Crustacea: Decapoda: Brachyura): a link between the Bythograeoidea and Xanthoidea. *Zoologica Scripta*. **39**, 621 – 630, doi: 10.1111/j.1463-6409.2010.00442.x (2010).
42. Yu, Y.Q., Ma, W.M., Yang, W.J., Yang, J.S. The complete mitogenome of the lined shore crab *Pachygrapsus crassipes* Randall 1840 (Crustacea: Decapoda: Grapsidae). *Mitochondrial DNA* **25**, 263–264, doi: 10.3109/19401736.2013.800497 (2014).
43. [!], Xing,Y., Ma, X., Wei, Y., Pan, D., Liu, W. Sun,H. The complete mitochondrial genome of the semiterrestrial crab, *Chiromantes neglectum* (Eubrachyura: Grapsoidea: Sesarmidae). *Mitochondrial DNA B Resour* **1**, 461-463, doi: 10.1080/23802359.2016.1186509 (2016).
44. [!], Wang, Y., Bai, J., Zhu, C.C., Zou, J.X. Zhou, X.M. **Unpublished**. *Huananpotamon lichuanense*

## *Maja crispata*; 16592 bp; MajGO

| | start | end | size | | |
|---|---|---|---|---|---|
| *cox1* | 1 | 1534 | 1534 | ATG | T(aa) |
| *trnL2* | 1535 | 1598 | 64 | taa | |
| isp (*trnL2-cox2*) | 1599 | 1604 | 6 | | |
| *cox2* | 1605 | 2292 | 688 | ATG | T(aa) |
| *trnK* | 2293 | 2359 | 67 | ttt | |
| *trnD* | 2360 | 2425 | 66 | gtc | |
| *atp8* | 2426 | 2584 | 159 | GTG | TAA |
| *atp6* | 2578 | 3251 | 674 | ATT | TA(a) |
| *cox3* | 3252 | 4041 | 790 | ATG | T(aa) |
| *trnG* | 4042 | 4105 | 64 | tcc | |
| *nad3* | 4106 | 4459 | 354 | ATT | TAA |
| isp (*nad3-trnA*) | 4460 | 4463 | 4 | | |
| *trnA* | 4464 | 4528 | 65 | tgc | |
| isp (*trnA-trnR*) | 4529 | 4531 | 3 | | |
| *trnR* | 4532 | 4594 | 63 | tcg | |
| *trnN* | 4595 | 4661 | 67 | gtt | |
| isp (*trnN-trnS1*) | 4662 | 4663 | 2 | | |
| *trnS1* | 4664 | 4729 | 66 | tct | |
| *trnE* | 4730 | 4794 | 65 | ttc | |
| isp (*trnE-trnT*) | 4795 | 4829 | 35 | | |
| *trnT* | 4830 | 4893 | 64 | tgt | |
| isp (*trnT-nad6*) | 4894 | 4933 | 40 | | |
| *nad6* | 4934 | 5442 | 509 | ATC | TA(a) |
| *cob* | 5443 | 6577 | 1135 | ATG | T(aa) |
| *trnS2* | 6578 | 6642 | 65 | tga | |
| isp (*trnS2-trnQ*) | 6643 | 7568 | 926 | | |
| *trnQ* | 7569 | 7637 | 69 | ttg | |

| | start | end | size | | |
|---|---|---|---|---|---|
| isp (*trnQ-trnC*) | 7638 | 7829 | 192 | | |
| *trnC* | 7830 | 7891 | 62 | gca | |
| *trnY* | 7892 | 7955 | 65 | gta | |
| isp (*trnY-trnH*) | 7956 | 8062 | 107 | | |
| *trnH* | 8063 | 8130 | 68 | gtg | |
| isp (*trnH-trnF*) | 8131 | 8132 | 2 | | |
| *trnF* | 8133 | 8197 | 65 | gaa | |
| *nad5* | 8198 | 9931 | 1734 | ATG | TAA |
| isp (*nad5-nad4*) | 9932 | 9935 | 4 | | |
| *nad4* | 9936 | 11309 | 1374 | ATG | TAG |
| *nad4L* | 11303 | 11602 | 300 | ATG | TAA |
| isp (*nad4L-trnP*) | 11603 | 11635 | 33 | | |
| *trnP* | 11636 | 11702 | 67 | tgg | |
| isp (*trnP-nad1*) | 11703 | 11809 | 107 | | |
| *nad1* | 11810 | 12763 | 954 | ATT | TAG |
| isp (*nad1-trnL1*) | 12764 | 12768 | 5 | | |
| *trnL1* | 12769 | 12834 | 66 | tag | |
| *rrnL* | 12835 | 14113 | 1279 | | |
| *trnV* | 14114 | 14185 | 72 | tac | |
| *rrnS* | 14186 | 14989 | 804 | | |
| CoRe | 14990 | 15321 | 332 | | |
| *trnI* | 15322 | 15386 | 65 | gat | |
| isp (*trnI-trnM*) | 15387 | 15405 | 19 | | |
| *trnM* | 15406 | 15471 | 67 | cat | |
| *nad2* | 15472 | 16478 | 1007 | ATG | TA(a) |
| *trnW* | 16479 | 16546 | 68 | tca | |
| isp (*trnW-cox1*) | 16547 | 16592 | 46 | | |

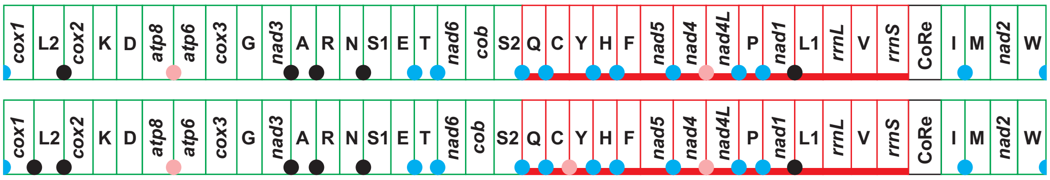


| | start | end | size | | |
|---|---|---|---|---|---|
| *cox1* | 1 | 1536 | 1536 | ATG | TAA |
| isp (*cox1-trnL2*) | 1537 | 1538 | 2 | | |
| *trnL2* | 1539 | 1602 | 64 | taa | |
| isp (*trnL2-cox2*) | 1603 | 1608 | 6 | | |
| *cox2* | 1609 | 2296 | 688 | ATG | T(aa) |
| *trnK* | 2297 | 2363 | 67 | ttt | |
| *trnD* | 2364 | 2428 | 65 | gtc | |
| *atp8* | 2439 | 2587 | 159 | GTG | TAA |
| *atp6* | 2581 | 3254 | 674 | ATT | TA(a) |
| *cox3* | 3255 | 4044 | 790 | ATG | T(aa) |
| *trnG* | 4045 | 4108 | 64 | tcc | |
| *nad3* | 4109 | 4462 | 354 | ATT | TAA |
| isp (*nad3-trnA*) | 4463 | 4466 | 4 | | |
| *trnA* | 4467 | 4530 | 64 | tgc | |
| isp (*trnA-trnR*) | 4531 | 4533 | 3 | | |
| *trnR* | 4534 | 4597 | 64 | tcg | |
| *trnN* | 4598 | 4664 | 67 | gtt | |
| isp (*trnN-trnS1*) | 4665 | 4666 | 2 | | |
| *trnS1* | 4667 | 4732 | 66 | tct | |
| isp (*trnS1-trnE*) | 4733 | 4733 | 1 | | |
| *trnE* | 4734 | 4798 | 65 | ttc | |
| isp (*trnE-trnT*) | 4799 | 4833 | 35 | | |
| *trnT* | 4834 | 4896 | 63 | tgt | |
| isp (*trnT-nad6*) | 4897 | 4936 | 40 | | |
| *nad6* | 4937 | 5445 | 509 | ATC | TA(a) |
| *cob* | 5446 | 6580 | 1135 | ATG | T(aa) |
| *trnS2* | 6581 | 6645 | 65 | tga | |
| isp (*trnS2-trnQ*) | 6646 | 7562 | 917 | | |

| | start | end | size | | |
|---|---|---|---|---|---|
| *trnQ* | 7563 | 7631 | 69 | ttg | |
| isp (*trnQ-trnC*) | 7632 | 7822 | 191 | | |
| *trnC* | 7823 | 7883 | 61 | gca | |
| *trnY* | 7883 | 7948 | 66 | gta | |
| isp (*trnY-trnH*) | 7949 | 8072 | 124 | | |
| *trnH* | 8073 | 8140 | 68 | gtg | |
| isp (*trnH-trnF*) | 8141 | 8142 | 2 | | |
| *trnF* | 8143 | 8207 | 65 | gaa | |
| *nad5* | 8208 | 9941 | 1734 | ATG | TAA |
| isp (*nad5-nad4*) | 9942 | 9951 | 10 | | |
| *nad4* | 9952 | 11319 | 1368 | ATG | TAG |
| *nad4L* | 11313 | 11612 | 300 | ATG | TAA |
| isp (*nad4L-trnP*) | 11613 | 11644 | 32 | | |
| *trnP* | 11645 | 11712 | 68 | tgg | |
| isp (*trnP-nad1*) | 11713 | 11820 | 108 | | |
| *nad1* | 11821 | 12777 | 957 | ATG | TAG |
| isp (*nad1-trnL1*) | 12778 | 12779 | 2 | | |
| *trnL1* | 12780 | 12845 | 66 | tag | |
| *rrnL* | 12846 | 14127 | 1282 | | |
| *trnV* | 14128 | 14199 | 72 | tac | |
| *rrnS* | 14200 | 15002 | 803 | | |
| CR | 15003 | 15339 | 337 | | |
| *trnI* | 15340 | 15404 | 65 | gat | |
| isp (*trnI-trnM*) | 15405 | 15435 | 31 | | |
| *trnM* | 15436 | 15503 | 68 | cat | |
| *nad2* | 15504 | 16510 | 1007 | ATG | TA(a) |
| *trnW* | 16511 | 16578 | 68 | tca | |
| isp (*trnW-cox1*) | 16579 | 16598 | 20 | | |

## *Maja squinado*; 16598 bp; MajGO

**Figure S1. The mitochondrial genomes of *Maja crispata* and *Maja squinado*.**
The MajGO gene order is depicted and linearized starting from cox1. Graphical representation of the mtDNAs and nomenclature of genes as in Figure 1. Isp, intergenic spacer; start, start of the genomic element; end, end of the genomic element; size, size of the genomic element. Start and stop are referred to the α-strand placement. For protein-coding genes, the start codon is provided in **blue** and the stop codon in **red** (with incomplete stop codons written in parentheses). The anticodon is provided (**purple**) for every tRNA (e.g., tga for *trnS2*). A **blue** circle marks an intergenic spacer associated to a genomic rearrangement (see main text). A **black** circle marks an intergenic spacer supposed to be the result of a DNA slippage, during the genome replication[1]. A **pink** circle marks an overlap between adjacent genes.

## The mtDNAs of *Maja crispata* and *Maja squinado*

In this study, the complete mtDNAs of the spider crabs *M. crispata* and *M. squinado* were sequenced and annotated. The final assembly of *M. crispata* was 16,592 bp long and contained 24,734 reads. Additional statistics for *M. crispata* mtDNA were: base coverage = 100%; mismatch = 0%; average coverage depth = 147.25; maximum coverage depth = 409. The final assembly of *M. squinado* was 16,598 bp long and contained 20,432 reads. The other statistics for *M. squinado* assembly were: base coverage = 100%; mismatch = 0%; average coverage depth = 121.50; and the maximum coverage depth was 227. The mtDNAs of M. crispata and M. squinado contain the full set of 37 genes found in metazoan mtDNAs (Fig. S1).

Both mtDNAs present intergenic spacers (isp in Fig. S1). These latter range from 1 base (*trnS1-trnE*, *M. squinado*) to 926 bases (*trnS2-trnQ*, *M. squinado*). The *trnS2-trnQ* intergenic spaces exhibit the largest size (Fig. S1). The newly determined mtDNAs share the same gene order MajGO, which is different from any other animal GO so far sequenced (Fig. S1). In MajGO, all the genes located on the β-strand form a single block, placed between *trnE* and CoRe. The A+T contents, the G+C contents, the AT-skews and GC-skews, as well as the codon usages of *M. crispata* and *M. squinado* mtDNAs fall within the ranges computed for the Brachyura mtDNAs (Supplementary Figs S2-S4). The 22 tRNAs are capable to produce the typical cloverleaf secondary structures (Supplementary Figs S5-S6). The *rrnL* and *rrnS* genes are also capable to fold in the secondary structures characterizing these genes. The *rrnS* and *rrnL* structures were determined through a homology modelling process, using as templates the several structures already available for crustacean and more in general for Arthropoda (e.g. Salvato *et al.*[1]; Babbucci *et al.*[2]). The detailed description of these structures will be presented in a paper dealing with the evolution of *rrnSs* and *rrnLs* of crustacean Decapoda, which is in preparation in our laboratory.

1. Salvato, P., Simonato, M., Battisti, A., & Negrisolo, E. The complete mitochondrial genome of the bag-shelter moth Ochrogaster lunifer (Lepidoptera, Notodontidae). *BMC Genomics* **9**, 331; 10.1186/1471-2164-9-331 (2008).
2. Babbucci, M., Basso, A., Patarnello, T., & Negrisolo E. Is It an ant or a butterfly? Convergent evolution in the mitochondrial gene order of Hymenoptera and Lepidoptera. *Genome Biol. Evol.* **6**, 326–3343; doi:10.1093/gbe/evu265 (2014).

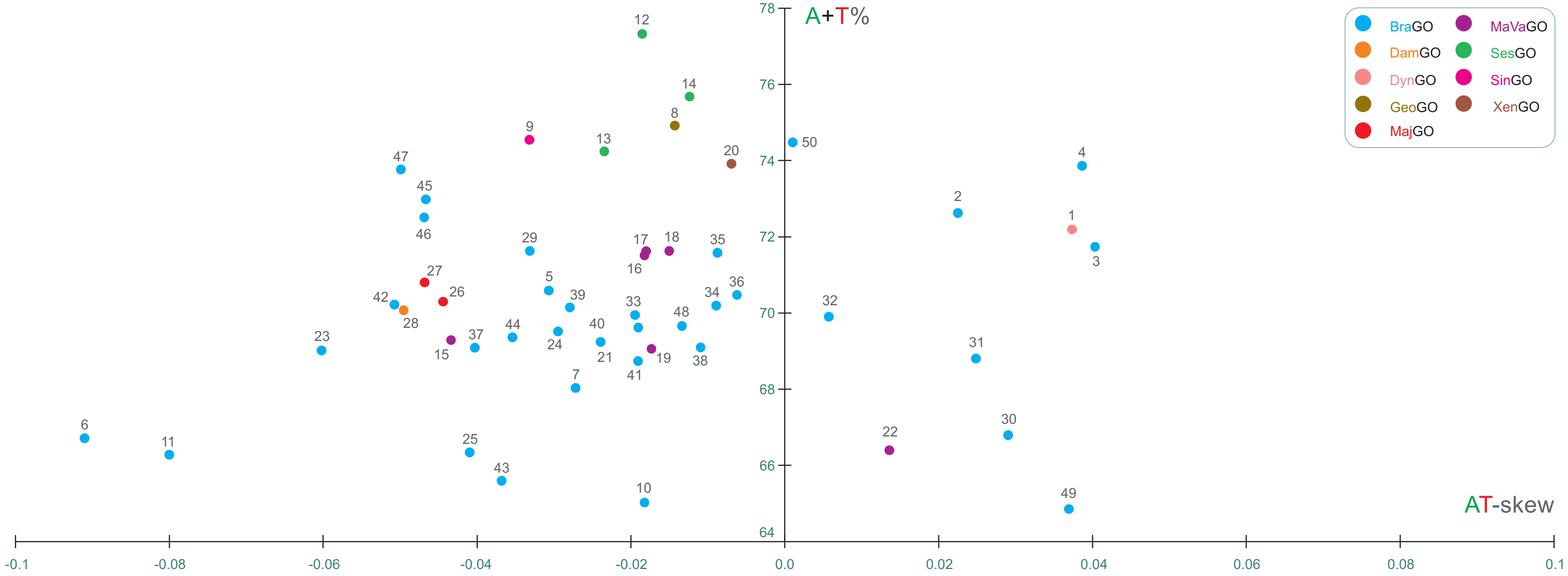


1 *Dynomene pilumnoides* Alcock, 1900
2 *Homola orientalis* Henderson, 1888
3 *Homologenus malayensis* Ihle, 1912
4 *Moloha majora* (Kubo, 1936)
5 *Lyreidus brevifrons* Sakai, 1937
6 *Ranina ranina* (Linnaeus, 1758)
7 *Umalia orientalis* (Sakai, 1963)
8 *Geothelphusa dehaani* (White, 1847)
9 *Sinopotamon xiushuiense* Dai, Zhou & Peng, 1995
10 *Grapsus tenuicrustatus* (Herbst, 1783)
11 *Pachygrapsus crassipes* Randall, 1840
12 *Metopaulias depressus* Rathbun, 1896
13 *Parasesarma tripectinis* (Shen, 1940)
14 *Sesarmops sinensis* (H. Milne Edwards, 1834)
15 *Cyclograpsus granulosus* H. Milne Edwards, 1853
16 *Eriocheir hepuensis* Dai, 1991
17 *Eriocheir japonica* (De Haan, 1835)
18 *Eriocheir sinensis* H. Milne Edwards, 1853
19 *Helice tientsinensis* Rathbun, 1931
20 *Xenograpsus testudinatus* N. K. Ng, Huang & Ho, 2000
21 *Ilyoplax deschampsi* (Rathbun, 1913)
22 *Macrophthalmus japonicus* (De Haan, 1835)
23 *Mictyris longicarpus* Latreille, 1806
24 *Ocypode ceratophthalmus* (Pallas, 1772)
25 *Ocypode cordimanus* Latreille, 1818
26 *Maja crispata* Risso, 1827
27 *Maja squinado* (Herbst, 1788)
28 *Damithrax spinosissimus* (Lamarck, 1818)
29 *Chionoecetes pacificus* Sakai, 1978
30 *Austinograea alayseae* Hessler & Martin, 1989
31 *Austinograea rodriguezensis* Tsuchida & Hashimoto, 2002
32 *Gandalfus puia* McLay, 2007
33 *Gandalfus yunohana* (Takeda, Hashimoto & Ohta, 2000)
34 *Ashtoret lunaris* (Forskål, 1775)
35 *Myomenippe fornasinii* (Bianconi, 1851)
36 *Pseudocarcinus gigas* (Lamarck, 1818)
37 *Chaceon granulatus* (Sakai, 1978)
38 *Callinectes sapidus* Rathbun, 1896
39 *Charybdis feriata* (Linnaeus, 1758)
40 *Charybdis japonica* (A. Milne-Edwards, 1861)
41 *Portunus pelagicus* (Linnaeus, 1758)
42 *Portunus trituberculatus* (Miers, 1876)
43 *Portunus sanguinolentus* (Herbst, 1783)
44 *Scylla olivacea (Herbst, 1796)*
45 *Scylla paramamosain* Estampador, 1949
46 *Scylla serrata* (Forskål, 1775)
47 *Scylla tranquebarica* (Fabricius, 1798)
48 *Thalamita crenata* Rüppell, 1830
49 *Leptodius sanguineus* (H. Milne Edwards, 1834)
50 *Pyrhila pisum* (De Haan, 1841)

**Figure S2. AT-skew vs. A+T% in the 50 Brachyuram mtDNAs.**
The values were calculated on the **α-strand** of the mtDNA genomes. The X axis provides the **AT-skew** values, while the Y axis provides the **A+T%** values.
**BraGO**, Brachyuran basic GO; **DamGO**, *Damithrax spinosissimus* GO; **DynGO**, *Dynomene pilumnoides* GO; **GeoGO**, *Geothelphusa dehaani* GO; **MajGO**, *Maja* genus GO; **MaVaGO**, Macrophthalmidae + Varunidae GO; **SesGO**, Sesarmidae GO; **SinGO**, *Sinopotamon xiushuiense* GO; **XenGO**, *Xenograpsus testudinatus* GO.

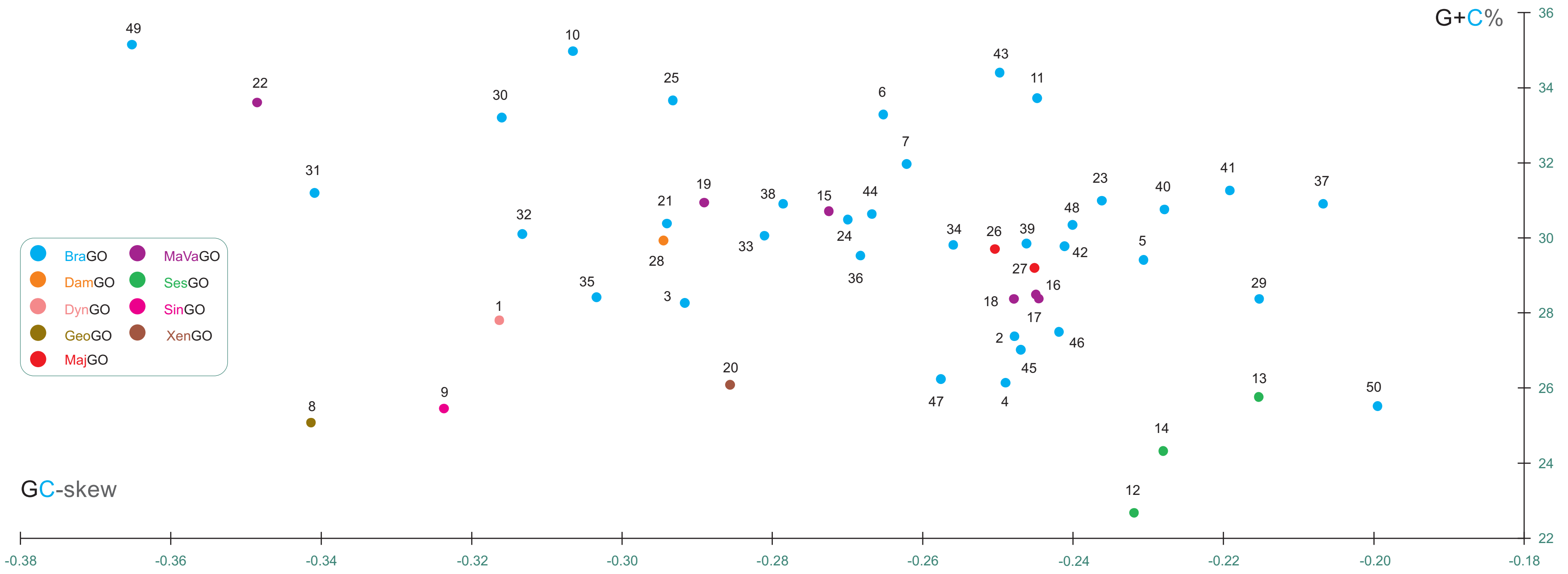


1 *Dynomene pilumnoides* Alcock, 1900
2 *Homola orientalis* Henderson, 1888
3 *Homologenus malayensis* Ihle, 1912
4 *Moloha majora* (Kubo, 1936)
5 *Lyreidus brevifrons* Sakai, 1937
6 *Ranina ranina* (Linnaeus, 1758)
7 *Umalia orientalis* (Sakai, 1963)
8 *Geothelphusa dehaani* (White, 1847)
9 *Sinopotamon xiushuiense* Dai, Zhou & Peng, 1995
10 *Grapsus tenuicrustatus* (Herbst, 1783)
11 *Pachygrapsus crassipes* Randall, 1840
12 *Metopaulias depressus* Rathbun, 1896
13 *Parasesarma tripectinis* (Shen, 1940)
14 *Sesarmops sinensis* (H. Milne Edwards, 1834)
15 *Cyclograpsus granulosus* H. Milne Edwards, 1853
16 *Eriocheir hepuensis* Dai, 1991
17 *Eriocheir japonica* (De Haan, 1835)
18 *Eriocheir sinensis* H. Milne Edwards, 1853
19 *Helice tientsinensis* Rathbun, 1931
20 *Xenograpsus testudinatus* N. K. Ng, Huang & Ho, 2000
21 *Ilyoplax deschampsi* (Rathbun, 1913)
22 *Macrophthalmus japonicus* (De Haan, 1835)
23 *Mictyris longicarpus* Latreille, 1806
24 *Ocypode ceratophthalmus* (Pallas, 1772)
25 *Ocypode cordimanus* Latreille, 1818
26 *Maja crispata* Risso, 1827
27 *Maja squinado* (Herbst, 1788)
28 *Damithrax spinosissimus* (Lamarck, 1818)
29 *Chionoecetes pacificus* Sakai, 1978
30 *Austinograea alayseae* Hessler & Martin, 1989
31 *Austinograea rodriguezensis* Tsuchida & Hashimoto, 2002
32 *Gandalfus puia* McLay, 2007
33 *Gandalfus yunohana* (Takeda, Hashimoto & Ohta, 2000)
34 *Ashtoret lunaris* (Forskål, 1775)
35 *Myomenippe fornasinii* (Bianconi, 1851)
36 *Pseudocarcinus gigas* (Lamarck, 1818)
37 *Chaceon granulatus* (Sakai, 1978)
38 *Callinectes sapidus* Rathbun, 1896
39 *Charybdis feriata* (Linnaeus, 1758)
40 *Charybdis japonica* (A. Milne-Edwards, 1861)
41 *Portunus pelagicus* (Linnaeus, 1758)
42 *Portunus trituberculatus* (Miers, 1876)
43 *Portunus sanguinolentus* (Herbst, 1783)
44 *Scylla olivacea (Herbst, 1796)*
45 *Scylla paramamosain* Estampador, 1949
46 *Scylla serrata* (Forskål, 1775)
47 *Scylla tranquebarica* (Fabricius, 1798)
48 *Thalamita crenata* Rüppell, 1830
49 *Leptodius sanguineus* (H. Milne Edwards, 1834)
50 *Pyrhila pisum* (De Haan, 1841)

**Figure S3. GC-skew vs. G+C% in the 50 Brachyuram mtDNAs.**
The values were calculated on the **α-strand** of the mtDNA genomes. The X axis provides the **GC-skew** values, while the Y axis provides the **G+C%** values.
**BraGO**, Brachyuran basic GO; **DamGO**, *Damithrax spinosissimus* GO; **DynGO**, *Dynomene pilumnoides* GO; **GeoGO**, *Geothelphusa dehaani* GO; **MajGO**, *Maja* genus GO; **MaVaGO**, Macrophthalmidae + Varunidae GO; **SesGO**, Sesarmidae GO; **SinGO**, *Sinopotamon xiushuiense* GO; **XenGO**, *Xenograpsus testudinatus* GO.

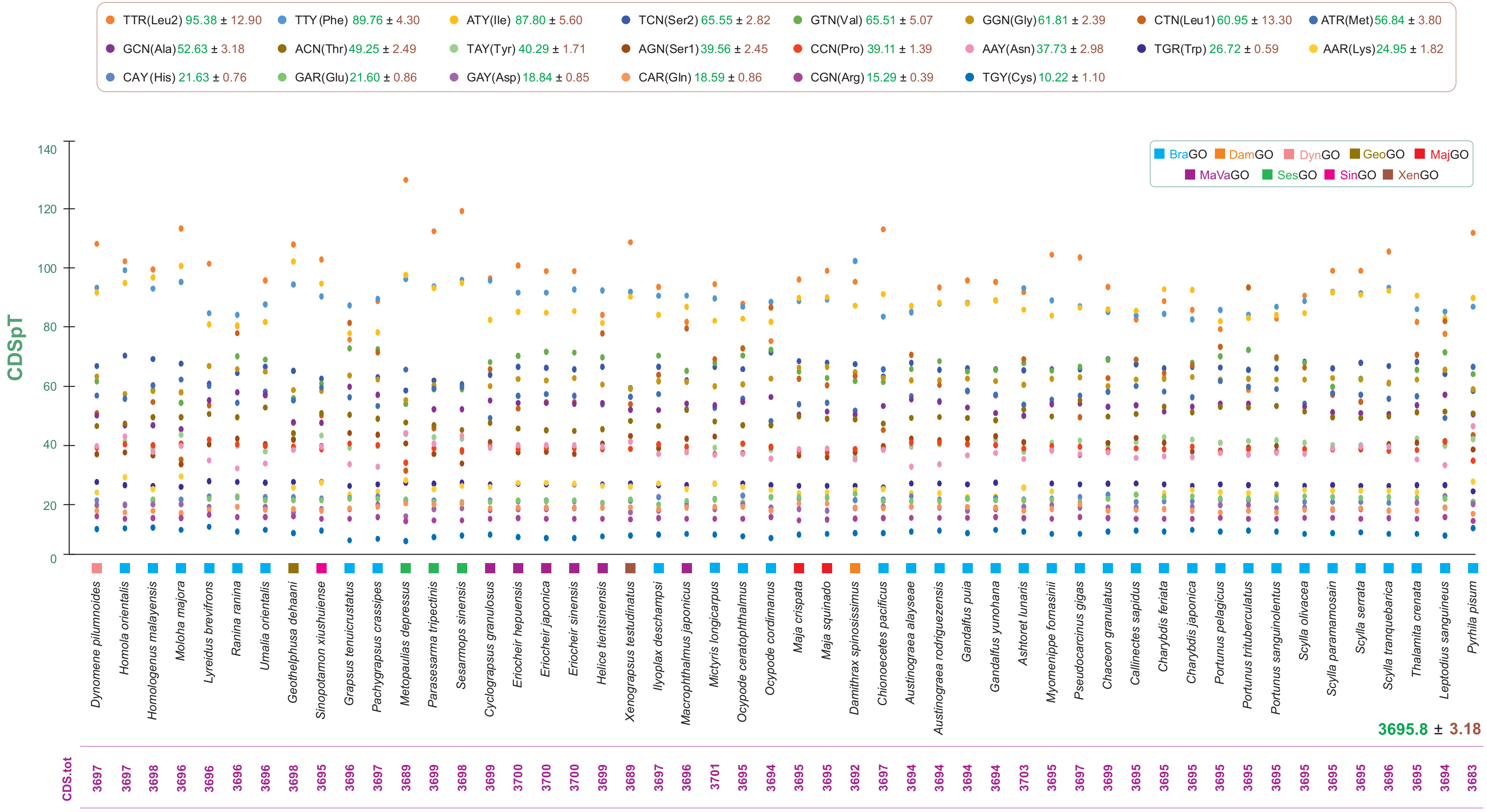


**Figure S4. Codon distribution in Brachyuran mtDNAs.**
**CDS.tot**, the total number of codons. **CDSpT**, codons per thousand codons. Codon families are provided in the inset above the graphic. The **average value** and the **standard deviation**, computed for the 50 Brachyuran mtDNAs, are provided for each codon family.
**Bra GO**, Brachyuran basic GO; **Dam GO**, *Damithrax spinosissimus* GO; **Dyn GO**, *Dynomene pilumnoides* GO; **Geo GO**, *Geothelphusa dehaani* GO; **Maj GO**, *Maja* genus GO; **MaVa GO**, Macrophthalmidae + Varunidae GO; **Ses GO**, Sesarmidae GO; **Sin GO**, *Sinopotamon xiushuiense* GO; **Xen GO**, *Xenograpsus testudinatus* GO.

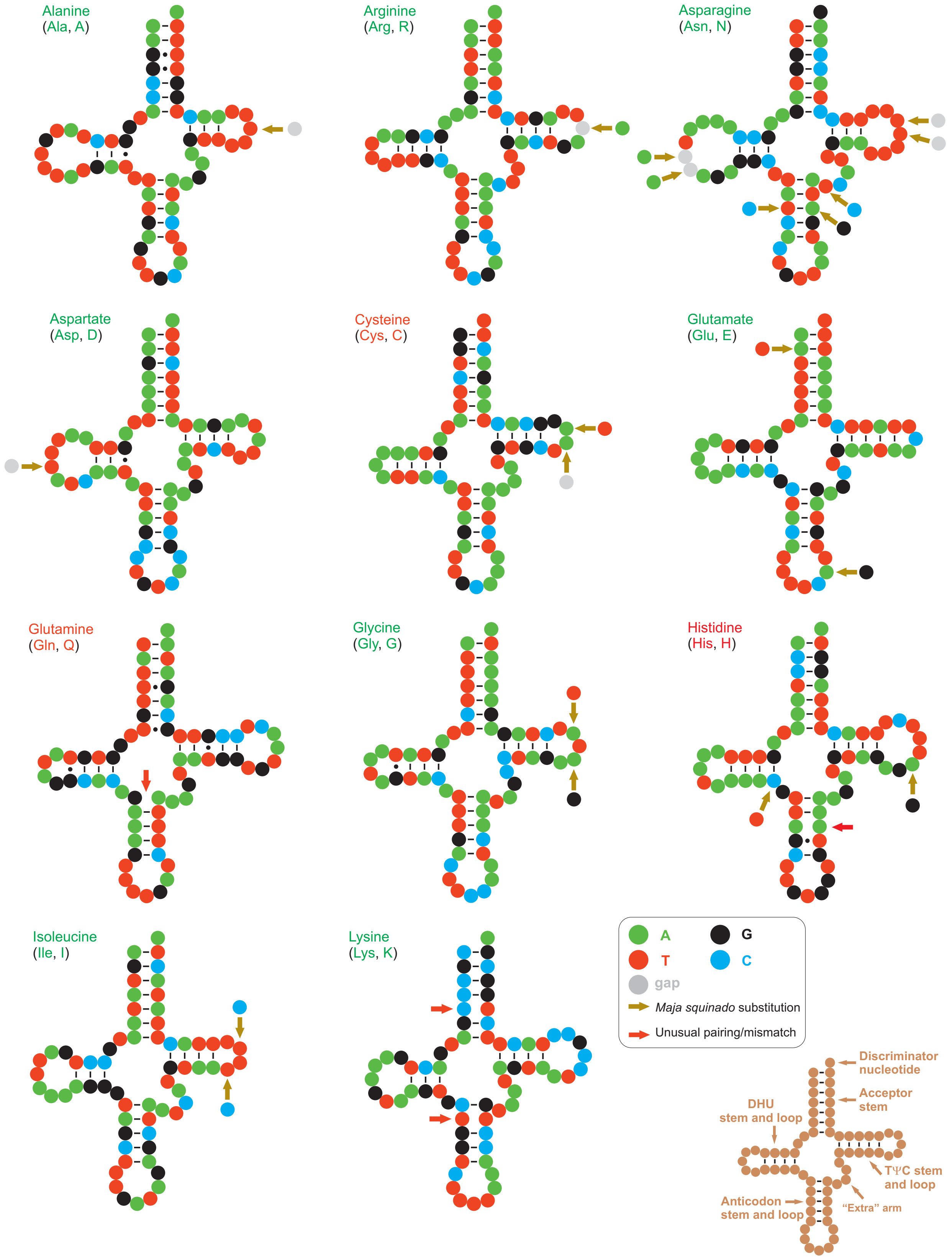


**Figure S5. Secondary structure of *Maja* tRNAs and level of conservation (*trnA*-*trnK*).**
Secondary structures are based on the tRNA sequences of *Maja crispata*. A substitution occurring in the same position for the tRNAs of *Maja squinado* is marked with a **light-brown** arrow. An unusual pairing/mismatch in the secondary structure is marked with a **red** arrow. A standard pairing is figured with a dash (-), while the G T pairing is figured wit a dot.

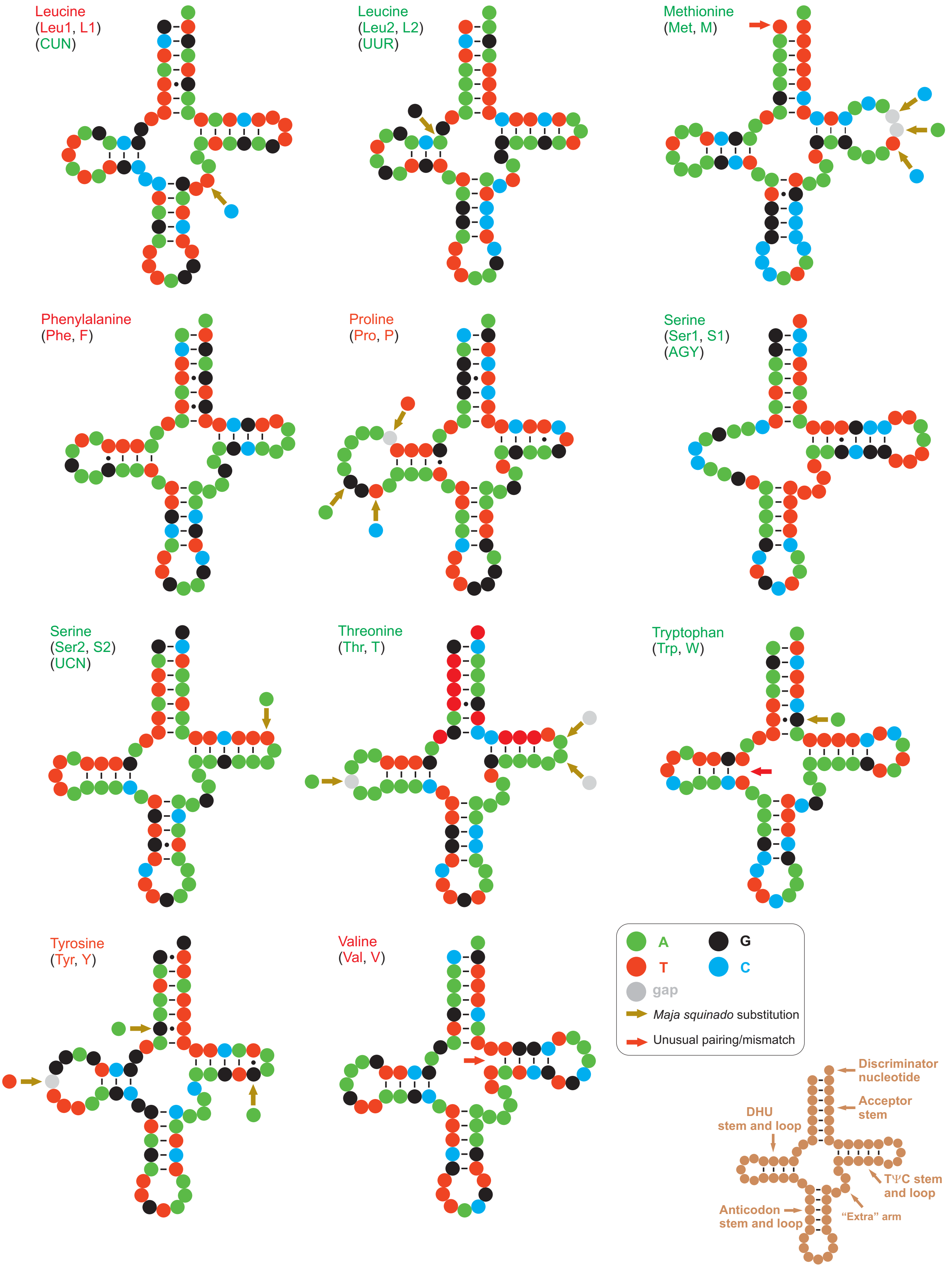


**Figure S6. Secondary structure of *Maja* tRNAs and level of conservation (*trnL1*-*trnV*).**
Secondary structures are based on the tRNA sequences of *Maja crispata*. A substitution occurring in the same position for the tRNAs of *Maja squinado* is marked with a **light-brown** arrow. An unusual pairing/mismatch in the secondary structure is marked with a **red** arrow. A standard pairing is figured with a dash (-), while the G T pairing is figured wit a dot.

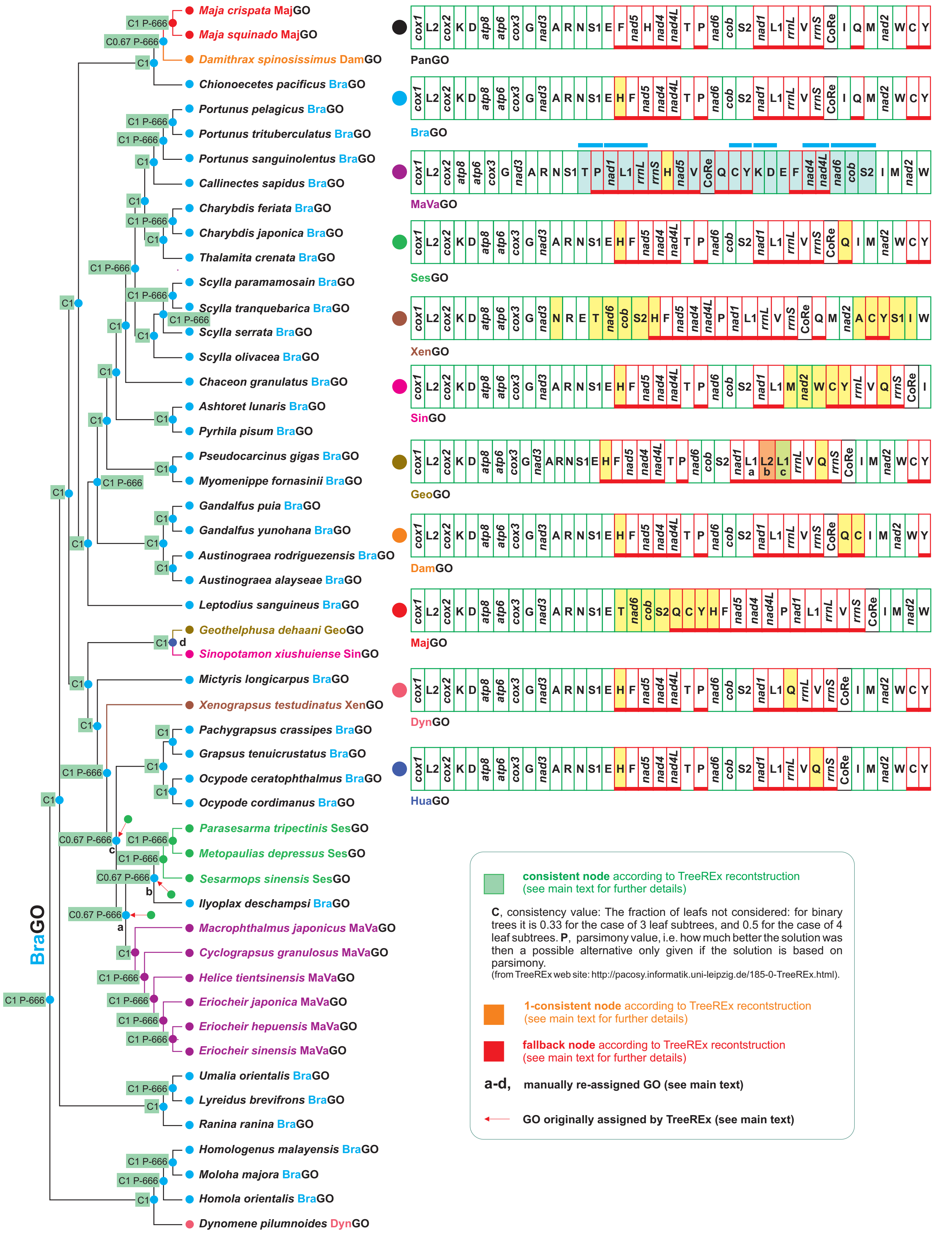


**Figure S7. Evolution of GOs in Brachyura: the TreeREx outputs.**
The GO assigned at each node by TreeREx is presented together with the associated score (**consistent**, **1-consistent**, **fallback** node). **Green square**, consistent node. **Orange square**, 1-consistent node. **Red square**, fallback node. The different colours reflect the level of uncertainty that characterises the reconstruction of the GO. The red squares indicate the highest level of uncertainty with respect to alternative GOs, while orange and particularly green squares point to more reliable GO reconstructions. (See main text for details).
The genes that changed their position relative to PanGO, through a transposition event, are shown with a yellow background. The passively-shifted genes are figured with their original background. The genes involved in a repositioning, which cannot be identified unambiguously as the result of a transposition or a passive shift, are figured with a light blue background. In this latter case, the common intervals, encompassing two or more genes, shared by the re-arranged GO with PanGO, are highlighted with an upper light blue bar.

**Figure S8. Pairwise-alignments of genes involved in genomic rearrangements and the associated intergenic spacers.**
The pairwise-alignments were performed with the ClustalW program, available at the PRABI/Rhone-Alpes Bioinformatics Center (https://npsa-prabi.ibcp.fr/cgi-bin/npsa_automat.pl?page=/NPSA/npsa_server.html). Successively, the alignments were manually improved through visual inspection. rc_sequence_name = reverse complement sequence of a gene encoded in the β-strand

**Figure S8.a**

# MajGO

## Intergenic spacer: ISP_trnT_nad6 (see Figure 5, main text)

```
                                   10        20        30        40        50        60      69
                                    |         |         |         |         |         |
Maja_crispata_ISP_trnT_nad6    ---------TCAGAGTTCT-T-TGTA-GTCTCCAAATTATAATACCTCTGGT-----------------
rc_Maja_crispata_trnP          TCAGAGAAAGAAGACTTCTATCTTAACTC-CCAAAGT-TAATATTTTACCTTTTTAAACTATTCCCTG

                                   10        20        30        40        50        60
                                    |         |         |         |         |         |
Maja_squinado_ISP_trnT_nad6    ---------TCAGAGTTCT---TGTAA-TCTCCAAATTTGTAGTACCTCTGGT----------------
rc_Maja_squinado_trnP          TCAGAGAAAGAAGACTTCTATCTTAACTC-CCAAAGTTAATATTTTGCTTTTTTAAAACTATTCCCTG
```

## Intergenic spacer: ISP_trnS2_trnQ (see Figure 5, main text)

```
                                   10        20        30        40        50        60        70        80        90       100
                                    |         |         |         |         |         |         |         |         |         |
Maja_crispata_ISP_trnS2_trnQ    TTTATATATTTATATACTATATTTTCTTATATAATATATATATATTACTTAAACCTCTTATAAAATTAAATAGTTATACAGATACAATACAAAATAATTA
rc_Maja_crispata_trnL1          ------------------TACTA-CTTATAGAAAAC-TCTATTTAACTAGAACCT------AAATCTAG-GGC-ATATA-AT-CTGC-CAAAATAGC--

                                  210       220       230       240       250       260       270       280       290       300
                                    |         |         |         |         |         |         |         |         |         |
Maja_squinado_ISP_trnS2_trnQ    TATGGACACATTACTTATACACACTTAAAATTATAAGTTACAAATAAATCTTTAAACACTTGGAATAAATATAAACTATTATAAACAACTCTATGCAAGT
rc_Maja_squinado_trnL1          --------TACTACTTATAG------AAAACTCTA--TT-TGACTAGAACC-TAAAT-CTAGG---CATATAATCTGCCA-AAATAGC-----------

                                      150       160       170       180       190       200       210       220       230
                                        |         |         |         |         |         |         |         |         |
Maja_crispata_ISP_trnS2_trnQ        TTTCCAAAATTCCTTACAACAACAATTTATTCAACAAGTTTATTTTCTACACCATAGAAATGCAGACATTCAATTTT-AAACCTAACTTCT
rc_Maja_crispata_trnV               ---TCAAAGT-----A-AAT--CGATTTGCACGATAA-TTT-TTTTC-A-AC-GTA--AACG-AAATGCTTAACTTTCAAGCTCTACTTTG

                                  580       590       600       610       620       630       640       650       660
                                    |         |         |         |         |         |         |         |         |
Maja_squinado_ISP_trnS2_trnQ    GTGTATCTTAAATTAAAATTTATTATCCTACTATAATTTTTTT-----TAGATCAAATGCCTTTATTCCAGGCTCAATGTTTCTTTCCC
rc_Maja_squinado_trnV           -------TCAAAGTAAA-TCGATT-TGC-AC-GATAATTTTTTTCAACGTAAACGAAATGCTTAACTTTCAAGCTCTAC-TTTG------

                                           780       790       800       810       820       830       840       850
                                             |         |         |         |         |         |         |         |
Maja_crispata_ISP_trnS2_trnQ            ACTATAAAGACCTC-TTAATTAGAGTACCCTACTTCCATATAGTTAACATCTAGTAAATTATATTTAACATTATTTTATA
Maja_crispata_trnI                      AGTATAATG-CCTGATTAAA-AGGGTA----GCTTTGATAGAGCTA--ATCATGTAA-TT-T-TTTA-C-TTATACTA--

                                  760       770       780       790       800       810       820       830       840
                                    |         |         |         |         |         |         |         |         |
Maja_squinado_ISP_trnS2_trnQ    CTACTTCGACTATAAAGACCTCAAAATTAGAGTACTTATTTGGATGTACCTAA-CATCTAATAACTTATACTTAATA-TAGAATTATAC
Maja_squinado_trnI              --------AGTATAATG-CCTGATTAAAAGGGTAGCTT-T---GATAGAGCTAATCATGTAACT-CTT-TACTTA-TACTA---------
```

## Intergenic spacer: ISP_trnQ_trnC (see Figure 5, main text)

```
                                   10        20        30        40        50        60        70        80        90       100
                                    |         |         |         |         |         |         |         |         |         |
Maja_crispata_ISP_trnQ_trnC    CCTTAAACTGCACCGTTTATAGATCAGCTCTTTGAATCATAGACAGCTGTGCCGCCTATCTTTAAAGCAAAAAAAAAAATTTTTCTTAAGGTTTGATTT
Maja_crispata_trnM             ----------------TAAAGATAAGCT-----AATAAAACTAA-TGGGCT-CATACCCCGTAATGAGAAATACACTCTCTTTTAA----------

                                  10        20        30        40        50        60        70        80        90       100
                                   |         |         |         |         |         |         |         |         |         |
Maja_squinado_ISP_trnQ_trnC    TTTTAAGCATATGCCATTATAGGCCTCATCTTTGAGCCAAGATAGCTTGTACTCAGCTACCTTAAAAACAAAAAAAATTATTTTTCTAAGTTTGGTTT
Maja_squinado_trnM             --TAAAGATAAGCTA--ATAAA-----------AGCTAATG-GGCTCATACCCCG-TAAATGAGAAACACAC----ACT-CTCTTTTA----------

                                  110       120       130       140       150       160       170       180       190
                                    |         |         |         |         |         |         |         |         |
Maja_crispata_ISP_trnQ_trnC    TTCTAATTTTTTTTTTAAGAATTTTATTTTATACTTATTATAAATCCTTTGACTTAATTTTTACACCTATTTCTTTAATTAATATCATTAAT
Maja_crispata_trnW             -----------------AGAATTTTATGTTATTCAAACTA-AAAGCCTTCAA---AGCTTTCGAAAAAGTATACTCTTTTAGATT-CTA--

                                  110       120       130       140       150       160       170       180       190
                                    |         |         |         |         |         |         |         |         |
Maja_squinado_ISP_trnQ_trnC    TTTCTAATTTTTTTTTTTAGAATTTTATTTTACAC---TTAGATAGATCACAAAGTCAACTTAATGATTATTCCTTTTATTGATCCATAAAATA
Maja_squinado_trnW             ------------------AGAATTTTATGTTATTCAAACTAAA-AGCCTTCAAAGCTTTCGAAAAAGTATACTCTTT-TAAATTC-TA-----
```

## Intergenic spacer: ISP_nad4L_trnP (see Figure 5, main text)

```
                                   10        20        30        40        50        60
                                    |         |         |         |         |         |
Maja_crispata_ISP_nad4L_trnP    -----------------------TTG-T-TT-TAAATATAAAATCATAAA--TCTACTGTC---
Maja_crispata_trnT              GTTTTAGTAGTTTAAAAAAAACTTTGGTCTTGTAAACCAAAATGAAAAATTTTCCTGAAACT

                                   10        20        30        40        50        60
                                    |         |         |         |         |         |
Maja_squinado_ISP_nad4L_trnP    -------TTGTTTAAAATA-----------TG-AAATCATAAATTACTATC-----------
Maja_squinado_trnT              GTTTTAGTAGTTTAAAAAAAACTTTGGTCTTGTAAACCAAAAATGAAAATTTTCCTGAAACT
```

## Figure S8.b

# MajGO

### Intergenic spacer: ISP_trnP_nad1 (see Figure 5, main text)

```
                                   10        20        30        40        50        60        70        80        90       100
                                    |         |         |         |         |         |         |         |         |         |
Maja_crispata_ISP_trnP_nad1   GACTATATTACATGTCTCTAGTTAATCATTTATTCAACATAGTAT-TTAATCAAACCAAATACAAATATTTATATATAATTAATTTTAAACTTTCATAGT
Maja_crispata_trnS2           ----------GAT-TATTTAGTT--T-ATTTAA--AACAT-GTGTCTTGAAAATACAAGAAAGAAAATTTCTTAATAATCG------------------

                                   10        20        30        40        50        60        70        80        90       100
                                    |         |         |         |         |         |         |         |         |         |
Maja_squinado_ISP_trnP_nad1   TGACTAATATTATTATTTTGATTATCATTTATTTATATGTCATTTTTAGTTAAACCGAATATAAATATTAATATATATTAATTAATTTTGAACTTTTATGAT
Maja_squinado_trnS2           ----------GATTATTTAGTTTAT--TTAAAACATGTGTC-TTGAAAATACAA--GAAAAGAAAAATT--CTTAATAATCG------------------


                                  160       170       180       190       200       210       220       230       240       250
                                    |         |         |         |         |         |         |         |         |         |
Maja_crispata_ISP_trnP_nad1   --GACTATA-TTAC-ATGTCTCT---AGT--TAATCA-TTTATTCA---ACATAG--TATTTA--TCAAACCAAATA----CA-----AATATTTATAT
Maja_crispata_nad6            TATATCTTGTTTATAATTTTTCTTGGAGGAATACTCGTTTTATTCATTTACGTATCCTCTTTAGCTTCCAACGAAATTTTTTCATTTTCAGCCTCTAGAT

                                  260       270       280       290       300
                                    |         |         |         |         |
Maja_crispata_ISP_trnP_nad1   --ATAATTAATTTTAAACTT-TCA-TAGTATTATTTT-------------
Maja_crispata_nad6            TTATAGTTA-TTTTAACCTTGTCACTAATTTTATTTTTCTTATTTATATT


                                  130       140       150       160       170       180       190       200       210       220
                                    |         |         |         |         |         |         |         |         |         |
Maja_squinado_ISP_trnP_nad1   -TGACTAATA-TTATT-ATTTTGATTATCATTTAT-TTATATGTCATTTTTAGTTAAACCGAATATAAATATTAATATATTTATGTATCCTCTTTAGCTT
Maja_squinado_nad6            GGAATTTACACTTATTCATTTTGATTTTCATATATCTTATTTATAATTTTTC-TTGGGG-GAATACTCGT-TTTATTCATTAAT-TAA--TTTTGAACTT

                                  230       240
                                    |         |
Maja_squinado_ISP_trnP_nad1   TTA-TGATATTATTTT----
Maja_squinado_nad6            CAAACGAAATTTTTTCTTTT
```

### Intergenic spacer: ISP_trnI_trnM (see Figure 5, main text)

```
                                   10        20        30        40        50        60
                                    |         |         |         |         |         |
Maja_crispata_ISP_trnI_trnM   ------------AAGTT-TA--A--GCTT----GAT-AAAA----------------------------
rc_Maja_crispata_trnQ         TTATCTGCAACGGAGTTACACCATTACTTTAAAGATCAAAACTTTCTGTGCGCTATACACCAACAAATA

                                   10        20        30        40        50        60
                                    |         |         |         |         |         |
Maja_squinado_ISP_trnI_trnM   AAATTTTAAACTTAA--ACCCCAGTATTTTAAT------------------------------------
rc_Maja_squinado_trnQ         TTATCTGCAACGGAGTTACACCATTACTTTAAAGATCAAAACTTTCTGTGCGCTATACACCAACAAATA
```

rc = reverse complement sequence of a gene encoded in the β-strand

### Intergenic spacer: ISP_trnW_cox1 (see Figure 5, main text)

```
                                   10        20        30        40        50        60
                                    |         |         |         |         |         |
Maja_crispata_ISP_trnW_cox1   TAATAACGAGCTCTCTATAACGTTTGTTTCTTCATTCTCCACCCTT--------------------
rc_Maja_crispata_trnY         CAATAAAAAAGTATTTACT-TGTTTGTAGATTTTACAATCTACCGCTTAAACCTCAGCCATCTTATC

                                   10        20        30        40        50        60
                                    |         |         |         |         |         |
Maja_squinado_ISP_trnW_cox1   TAATAAC-AAGC-TCCACCC-TT-------------------------------------------
rc_Maja_squinado_trnY         CAATAAAAAAGTATTCACTTGTTTGTAGATTTACAATCTACCGCTTAAAACCTCAGCCATTTTATC
```

# DamGO

### Intergenic spacer: ISP_trnQ_ trnC (see Figure 5, main text)

```
                                           10        20        30        40        50        60
                                            |         |         |         |         |         |
Damithrax_spinosissimus_ISP_trnQ_trnC   ----------------TATAAAATATT-AGCCTCGAACTCTTT-------------------------
Damithrax_spinosissimus_trnW            AGGATTTTAAGTTATTTATTTAAACTTAAAGCCTTCAAAGCTTTCAAAAAAAGATTATTTTAAATCCTA
```

### Intergenic spacer: ISP_trnI_ trnM (see Figure 5, main text)

```
                                             10        20        30        40        50        60
                                              |         |         |         |         |         |
Damithrax_spinosissimus_ISP_trnI_trnM   -----CCTGGTGTGATTTCATTCTTTCTAT--------------------------------------
Damithrax_spinosissimus_trnQ            TTATTTATTATGGAATTTAACCATACCTTAAAAAATCAAAGTTTTTCGTGCCCCTACACCAATAAATA
```

# HuaGO

### Intergenic spacer: ISP_trnV_trnQ (see Figure 6, main text)

```
                                                 40        50        60
                                                  |         |         |
Huananpotamon_lichuanense_ISP_trnV_trnQ   ----------AACAATATTA-AATATTTA-
Huananpotamon_lichuanense_CoRe            ATAATGAAATAAAAATATTATAATATTAAG
```

**Figure S8.c**

## GeoGO

### Intergenic spacer: ISP_trnL2_trnL1 (see Figure 6, main text)

```
                                    40        50        60        70        80        90       100       110
Geothelphusa_dehaani_ISP_trnL2_trnL1c   TTATTTAAATATTATAAATTAATTAACATGTAAAATTATAAATTTATAATATTTTATATAAATTTAAC-TAAATATACTTA
Geothelphusa_dehaani_trnL1a             -TATT---AT-TTATAAACCTAT--ATATATATA-TT-TAAATTC-TAA-ATTTAATGTAAACTTACATAAATA-AT---

                                   470       480       490       500       510       520       530       540
Geothelphusa_dehaani_ISP_trnL2_trnL1c   AAACACTACTTTTTTATAGTCTATTATATGTATATAAACCCCTAAATCCATTGATCAAGTCCCTATAATTAAAGTTAAAA
Geothelphusa_dehaani_trnL2              ------TA-TTACTTATAGACGAATCTATGTATTTAAATCT-TAAGTTTAATG--CAAA-CCTT---GCTAAAA-TAAC-

                                   470       480       490       500       510       520       530       540
Geothelphusa_dehaani_ISP_trnL2_trnL1c   AAACACTACTTTTTTATAGTCTATTATATGTATATAAACCCCTAAATCCATTGATCAAGTCCCTATAATTAAAGTTAAAA
Geothelphusa_dehaani_trnL1c             ---CACTACTTATAGATAA--GAATCTATATAATTAAAT-CCTAAATTTAATG--CAA--CCCT---GCCAAAGT-AAT-
```

### Intergenic spacer: ISP_cox1_cox2 (see Figure 6, main text)

```
                                      10        20        30        40        50        60        70
Geothelphusa_dehaani_ISP_cox1_cox2   -----AATAACAAATAATTGTATCTAGCTTTTATAACT-TTACAAAGAATTATATTTCTTTTAAAAATTTTA
Huananpotamon_lichuanense_trnL2      TCTAAAATGACAGAAAAT-GTATAGGACTTAAACCCCTATTATGAAGA-TTATTCTTCTTTTAGAA-------
```

## SinGO

### Intergenic spacer: ISP_trnS2_nad1 (see Figure 6, main text)

```
                                          120       130       140       150       160       170       180       190
Sinopotamon_xiushuiense_ISP_trnS2_nad1   AGTCCTTAACTTCAATAACA-ATAAACACAAATTTTAACTCATAAAATTTCTTAAAAACTAAAAACTATATATTATATTA
Sinopotamon_xiushuiense_trnS2            ATTATTTAGTTT-ATTAATTTATAAA-ACAAATGTT---TTG-AAAACA--TTAAAAAAGAAAGATTCT-TAATA-ATTA
```

### Intergenic spacer: ISP_trnL1_trnM (see Figure 6, main text)

```
                                       360       370       380       390       400       410       420       430       440
Sinopotamon_xiushuiense_ISP_trL1_trnM   ATCATAATATTTTATCATTTATTTATAATATAATATATTCTTAATTTGTTAATAAAACAATTATTAATTATATGTATATTATAATTTA
Sinopotamon_xiushuiense_trnV            -TCAAAGCAA---ATCG---ATTTGCACGATAAT-CAATTTCAA--TGTAAATGAAATA-CTATT--CTATTT-TA-GTTATACTTTG
```

## XenGO

### Intergenic spacer: ISP_trnS2_trnH (see Figure 7, main text)

```
                                                390       400       410       420       430       440       450       460
Xenograpsus_testudinatus_ISP_trnS2_trnH   --CTTCTATAAGCATGAATTATAACTAATCAATAGTAATTT--ATATT-ATAAAAC-ATTTTAAACTTATAAACTACC--
rc_Xenograpsus_testudinatus_nad1          ATCTTAAAGGAAAAGCAATTATAATAAATCAAAAATAACTTTGATATTCATTGAATAACTCTAAA-TTA-AAGCCTCCAA

                                                470       480       490       500       510       520       530       540
Xenograpsus_testudinatus_ISP_trnS2_trnH   TTATAAATTTATTTTACAAAAAAATTAGA---AATATTAA--CATACAATAATACTT-A-TAATTTCTATTATTAATCTA
rc_Xenograpsus_testudinatus_nad1          CTAATAACACAAAAGACAATAAAATTAAAGCTAATCTTACTTCATATGAAATTGTTTGAGCAACAGCTCGTATTCTTCCT

                                                550       560
Xenograpsus_testudinatus_ISP_trnS2_trnH   ATAAATTAAC--TTA-----
rc_Xenograpsus_testudinatus_nad1          AAAAGTGAATATTTACAATT
```

### Intergenic spacer: ISP_trnP_nad1 (see Figure 7, main text)

```
                                              640       650       660       670       680       690       700
Xenograpsus_testudinatus_ISP_trnP_nad1   --AATTTCTAT-AA-TTATATACA--TATAATATATTTATAATTTACATTTCCTGA-ATAATTA-ATTT-
Xenograpsus_testudinatus_cob             GGAATTTCAAGAAATTTAGATAAAGTTCCATTTCACCCATATTTTACATTTAAAGATATCGTCGGATTTC

                                              710       720       730
Xenograpsus_testudinatus_ISP_trnP_nad1   -A---ATAAAAACTATCTTCTTTTTA----
Xenograpsus_testudinatus_cob             TAGTTATATTAACTTTAGTTTTTTTATTAA
```

### Intergenic spacer: ISP_trnE_trnT (see Figure 7, main text)

```
                                         10        20        30        40        50        60        70        80        90
Xenograpsus_testudinatus_ISP_trnE_trnT   CCATTACTATAAGTCTCTTATAAACTAAATATTACTATTTTAACTACTTATA-AAATTTATAATAACT-TTTAACATTACCCTTA
rc_Xenograpsus_testudinatus_nad4L        CCAATACATTAAATCTCTTA-AAA-TAATCATTACCATGCCTGCGAACAATATAAATTAATAAGGATAACCCAACTACACCTTCA
```

**Figure S8.d**

## SesGO

### Intergenic spacer: ISP_trnQ_trnI (see Figure 7, main text)

```
                                          20        30        40        50        60        70        80        90       100
                                           |         |         |         |         |         |         |         |         |
Sesarma_neglectum_ISP_trnQ_trnI    CAATAAAGTTAATTAAATTTTAATGAATTCATTATAATATTATATATTAATAATTTATTGTAATTTTTAATTAT-TATATCATTTTCACA
Sesarma_neglectum_trnI             -AATATAGT-GCCT-GATTTAAAGGATA-GCTTTGA---TAGA-GCTAATC----A-TGTAATTTTT--TTACCTATATTA--------
```

```
                                          80        90       100       110       120       130       140       150
                                           |         |         |         |         |         |         |         |
Metopaulias_depressus_ISP_trnQ_trnI    AACATAATAT-TAACATAATATTAACATAATATTAACATAATATTAA-CATAATATTAACATAATATTAACATAATATTA
Metopaulias_depressus_trnI             AATATAGTGCCTGATTTAA-A--AGGATAGTGTTG--ATAGTGCCAATCATG-TATT----T--TTTTTACCT-ATATTA
```

```
                                              130       140       150       160       170       180       190
                                               |         |         |         |         |         |         |
Sesarmops_sinensis_KR336554_ISP_trnQ_trnI    ATAAAATTATTTTTATTATAAAATAAATAATTATATCAATTT-ATAATTTAATTTTTTCCACCAAACCCT
Sesarmops_sinensis_KR336554_trnI             --AATATAGTGCCTGATTTAAAAGGATAGCTTTGATAGAGCTAATCATGTAATTTTTTTACCTATATTA-
```

## DynGO

### Intergenic spacer: ISP_trnI_trnM (see Figure 8, main text)

```
                                            10        20        30        40        50        60
                                             |         |         |         |         |         |
Dynomene_pilumnoides_ISP_trnI_trnM    -------TAAT----TTTCA--ATTTTCCAAACAATAAAA--TTAT-------------
rc_Dynomene_pilumnoides_trnQ          ATATACGTAATGGAATTGCACCACCTCTTAAAAAATCAAACCTTTTCGTGCTTTCTACAC
```

### Intergenic spacer: ISP_trnL1_trnQ (see Figure 8, main text)

```
                                          230       240       250       260       270       280       290       300
                                           |         |         |         |         |         |         |         |
Dynomene_pilumnoides_ISP_trnL1_trnQ    ATTAAGTAATAATATCAATACAAATATATTAATACATTCATAATTTATTTAGGTCCTATTATATA--TACATTTAAAGAT
rc_Dynomene_pilumnoides_trnV           -TCAA--AATAA-ACCGATTTGCACG-ATTAATCCCCTCA-ACGTAACTGAGATGCTTCCTTATAGCTATATTTTG----
```

```
                                          340       350       360       370       380       390       400       410
                                           |         |         |         |         |         |         |         |
Dynomene_pilumnoides_ISP_trnL1_trnQ    AATATTATATTCTAAGAAAAATATAATAGTATATCGTTACAACTCAATTTTATAAACAGAGTAATCTTTCACAACAGTAA
Dynomene_pilumnoides_trnI              AATATAGTGC-CTGATAAAAAGG--GTAGC-T-TTGATAGAGCT-AA--TTATAAA--GTCCGACTTTCTATATTA----
```

## MaVaGO

### Intergenic spacer: ISP_nad4L_nad6 (see Figure, 8 main text)

```
                                             10        20        30        40        50        60        70        80
                                              |         |         |         |         |         |         |         |
Cyclograpsus_granulosus_ISP_nad4L_nad6    TAGTTTTAATAGTTTAGTTAAACCAACTAAAACATTGGTTTTTTACTCCTATGCCTGGAACTTTTCCTTAAACTAAAGAG
Cyclograpsus_granulosus_trnT              --GTTTTCATAGTTTA---AAAT---CCAAAACATTGGTCTTGTAAACCAATAATGAATAGATTTCTGTAAACT------
```

```
                                                 60        70        80        90       100       110       120       130       140
                                                  |         |         |         |         |         |         |         |         |
Macrophthalmus_japonicus_ISP_nad4L_nad6    ACAAATATATTTTTATAAAGAAAGTTTAACGTAAAAACTTTTTACATTGGTATCA-AAACCAAAGA-AAATAATCCATTTTAAACTTTAA
Macrophthalmus_japonicus_trnT              --------GTTTTTATA------GTTTAA----AAA-------ACATTGGTCTTGTAAACCGAAGATAAATCAGAT-TTTTAAAAACT--
```

### Intergenic spacer: ISP_trnP_nad1 (see Figure 8, main text)

```
                                             10        20        30        40        50
                                              |         |         |         |         |
Cyclograpsus_granulosus_ISP_trnP_nad1    -----------ACTATTTCGTTATTAACTT-TTTTAAACTTTAATTT--
Cyclograpsus_granulosus_nad6             ATTTTTATATTGACAATTCCTTTATTACTTTCTTTTTCATTTTTATTTAC
```

```
                                           10        20        30
                                            |         |         |
Helice_tientsinensis_ISP_trnP_nad1    -------AGTTAAC-TTTCCTT-ACTATCAT
Helice_tientsinensis_nad6             ATCTTTTTGTTAACAATTCCTTTTCTTTTAT
```

```
                                        190       200       210
                                         |         |         |
Eriocheir_hepuensis_ISP_trnP_nad1    -----AGTTT-ATTTACTCTAAATT-----
Eriocheir_hepuensis_nad6             ATATTAGTTTTATTCA-TTTATATTGCGTC
```

### Intergenic spacer: ISP_rrnL_rrnS (see Figure 8, main text)

```
                                            10        20        30        40        50        60        70
                                             |         |         |         |         |         |         |
Macrophthalmus_japonicus_ISP_rrnL_rnnS    ------------------------CATTAAAGACTTTATAACTAAGTTAA-ATTTTACTTTATAATT------
Macrophthalmus_japonicus_trnV             TCAAAGCAAACCGACTTGCACGGTAATTCTTCTCAATGTAACTGAGATACTATACTAATTTAGCTTTACTTTG
```

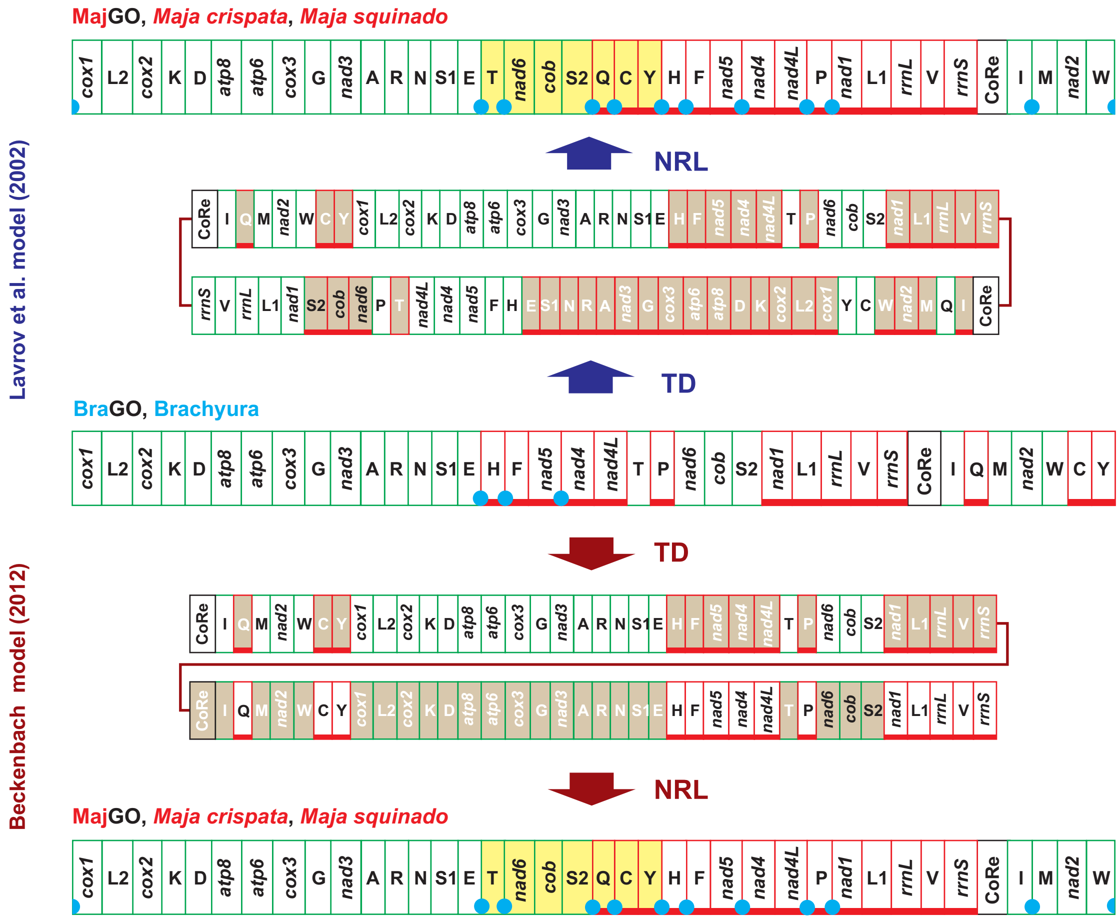


**Figure S9. The evolutionary pathways generating the MajGO modelled by tandem duplication nonrandom loss (TDNRL) models.**
The rearrangement in the GO of *Maja* species is investigated, and depicted with respect to **BraGO**. TD/NRL tandem duplication/ non random loss event. The genomic and genetic nomenclature, as well as the colour scheme, are the same as in Figure 1. The genes that changed their position relative to BraGO, through a transposition event, are shown with a yellow background. The passively-shifted genes are figured with their original background. A blue circle marks an intergenic spacer present in a position associated to genomic rearrangement (see Figure 5, main text).

### Text associated to Figure S9.

The MajGO arrangement can be explained by the tandem duplication nonrandom loss (TDNRL) models (Fig. S9). Two TDNRLs models exhist[1,2]. The model first was developed by Lavrov et al.[1] to explain the arrangement of two Diplopoda. In these millipedes most of the genes located in the same strand are contiguous, thus generating a strand-biased arrangement1. More recently, a second TDNRL model was presented by Beckenbach[2] to explain the genomic arrangement of the winter crane fly *Paracladura trichoptera*, which exhibits a GO with a stand-biased distribution of the genes, different however than that described for millipedes. Both models implied the duplication of the complete mtDNA. This process generates a dimeric molecule where the monomers are covalently linked head to tail (Lavrov et al.[1]) or head to head (Beckenbach[2]). For reason of space only the TDNRL model of Beckenbach[2] is presented in Figure 5 of main text.
The model of Lavrov et al.[1] implies the occurrence of a single, bidirectional, transcription promotor located in the CoRe, while the model of Beckenbach[2] assumes the presence of multiple transcription promotors located on both strands and associated with block of genes.
Both TDNRLs imply that, after the complete duplication of the mtDNA, the transcriptional promotor/s, which are located in the same strand, are lost/inactivated in the first monomer and the same process occurs in the opposite strand of the second monomer.
Thus, the genes, located in the strand deprived of their promotors, cannot be transcribed. This effect rapidly transforms the genes in pseudogenes, which are lost at the end. The final rearrangement is thus strand-biased and non random. However, no-biased transposition/inversion can also occur. The model of Lavrov et al.[1] explicitly predicts the presence of a second intergenic large spacer (indeed the duplication of the CoRe), while the model of Beckenbach[2] does not make any assumption on the presence/absence of intergenic spacers. Finally, none of the two TDNRL models denies the possibility that intergenic spacers are present in positions different than CoRe as the effect of the TDNRL process.
In *Maja* species, intergenic spacers of variable size (Supplementary Figs. S1, S9) are present in all the positions associated with genomic rearrangements. However, the presence of these spacers does not allow to decide what model (TDRL or TDNRL) describes better the evolutionary pathway that generated the *Maja* GO. Indeed the presence of these spacers is consistent with both models.

### References

1. Lavrov, D.V. Boore, J.L., & Brown, W.M. Complete mtDNA sequences of two millipedes suggest a new model for mitochondrial gene rearrangements: duplication and nonrandom loss. *Mol. Biol. Evol.* **19**, 163–169 (2002).
2. Beckenbach, A.T. Mitochondrial genome sequences of Nematocera (Lower Diptera): evidence of rearrangement following a complete genome duplication in a Winter Crane Fly. *Genome Biol. Evol.* **4**, 89–101; doi:10.1093/gbe/evr131 (2012).

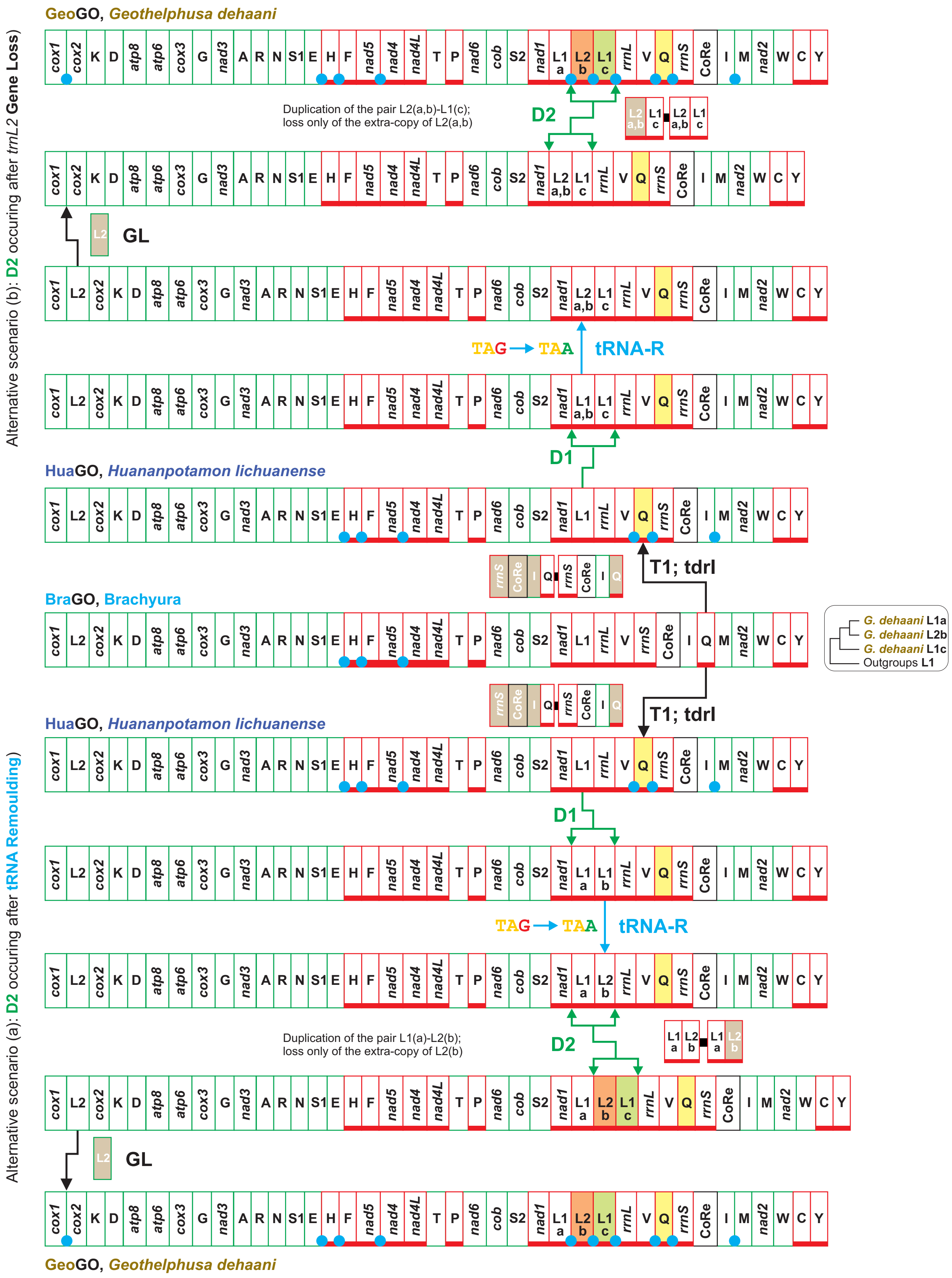


**Figure S10. Alternative transformational pathways for GeoGO.**
The rearrangements in the GOs of Potamid species are investigated and depicted with respect to BraGO. **T1**, transposition event; tdlr, duplication random loss, mechanism producing the observed re-arrangement; **D1-D2**, gene duplication events; **tRNA-R**, tRNA remoulding event; **GL**, gene loss event. The genomic and genetic nomenclature, as well as the colour scheme, are the same as in Figure 1 of main text.
